# The number of central nervous system-driven symptoms predicts subsequent chronic primary pain: evidence from UK Biobank

**DOI:** 10.1016/j.bja.2024.12.009

**Published:** 2025-01-27

**Authors:** Eoin Kelleher, Chelsea M. Kaplan, Dorna Kheirabadi, Andrew Schrepf, Irene Tracey, Daniel J. Clauw, Anushka Irani

**Affiliations:** 1 Nuffield Department of Clinical Neurosciences, University of Oxford, Oxford, UK; 2 Chronic Pain and Fatigue Research Center, University of Michigan, Ann Arbor, MI, USA; 3 Department of Rheumatology, Mayo Clinic, Jacksonville, FL, USA

**Keywords:** chronic pain, epidemiology, musculoskeletal pain, nociplastic pain, primary pain

## Abstract

**Background:**

Chronic primary pain describes conditions where pain is the principal problem rather than a consequence of another disease. Primary pain is thought to be primarily owing to nociplastic pain (i.e. pain as a result of altered nociception despite the absence of tissue damage). Primary pain is often accompanied by other bothersome central nervous system (CNS)-driven symptoms, including disturbed sleep, mood, and cognition; however, it is unclear whether these symptoms precede onset of primary pain.

**Methods:**

In a prospective cohort study of the UK Biobank, we examined adults with no self-reported recent or chronic pain at baseline. Using linked primary care record data, we investigated the association between the number of CNS-driven symptoms and subsequent incidence of primary pain conditions. Multivariable regression analyses adjusted for sociodemographic and lifestyle factors.

**Results:**

Of 502 369 participants, 70 630 (14.0%) met the inclusion criteria, with a mean (range) age of 56.7 (40-70) yr, 51% being female. After 7.4 (range 0.5–11.02) yr, 12.2% developed a primary pain condition. We observed a positive relationship between the number of CNS-driven symptoms at baseline and risk of future primary pain (HR 1.43, 95% CI 1.34–1.52, *P*<0.001). Participants with more CNS-driven symptoms at baseline were also more likely to have chronic and more severe nociplastic pain, but not non-nociplastic pain at follow-up.

**Conclusions:**

In adults with no current self-reported pain, those with a greater number of CNS-driven symptoms at baseline were more likely to develop a primary pain condition. This suggests a potential opportunity for early intervention in mitigating the burden of primary pain.

**Editor's key points**

• Primary pain includes conditions such as fibromyalgia, chronic low back pain, or irritable bowel syndrome. These patients often report central nervous system (CNS) symptoms and mood disorders, but it is unclear whether these symptoms precede or increase the risk of developing primary pain.
• In a prospective cohort study from the large UK biobank, authors show that a greater number of CNS symptoms (sleep, affect, cognition) in adults without pain at baseline is associated with increased risk of developing chronic primary pain but not other types of pain over a 10-yr period.
• This indicates the need for early recognition and intervention for these CNS symptoms.

Chronic pain affects up to half the global population, contributing significantly to global disability and ill health.^1^ The recent International Classification of Diseases 11th Revision (ICD-11) classification for the first time describes chronic primary pain as a diagnosis in its own right, where pain is the principal problem rather than a consequence of another disease.^2^^,^^3^ Primary pain is exemplified by conditions such as fibromyalgia, chronic low back pain, endometriosis, irritable bowel syndrome, and temporomandibular syndrome. These conditions are also collectively referred to as chronic overlapping pain syndromes (COPCs).^4^ The predominant pain mechanism in these conditions is thought to be nociplastic pain, a relatively recent mechanistic category of pain generalised by dysfunctional pain pathways resulting in widespread pain and sensory hypersensitivity.^5^ Analysis of primary pain disorders reveals two symptom clusters: generalised sensory sensitivity and SPACE (sleep, pain, affect, cognition, energy/fatigue),^6^ indicating a potential shared central nervous system (CNS) dysfunction in chronic primary pain. Patients with primary pain conditions frequently report CNS-driven symptoms including fatigue, insomnia, dyscognition (such as ‘fibro-fog’ in fibromyalgia), and mood disturbance.^7^ However, it remains unclear whether these symptoms precede onset of chronic primary pain disorders in adults, and if the burden of these symptoms increases the risk of developing a chronic primary pain condition.

In this study, we investigate the relationship between the burden of three CNS symptoms from the SPACE cluster (sleep, mood, and cognitive disturbance) and future primary pain. We hypothesise that adults with no chronic pain with a greater burden of SPACE symptoms will be at a higher risk of developing a primary pain disorder in the future.

Given that many primary pain conditions embody characteristics of nociplastic pain, we also examine the association with future pain phenotype and nociplastic pain severity. We hypothesise that a greater burden of these symptoms is associated with a greater risk of nociplastic-like, but not non-nociplastic-like, pain at follow-up.

## Methods

### Study design and setting

This study uses UK Biobank, a multicentre cohort study comprising British adults aged 40–69 yr registered with the NHS and living within 25 miles of one of 22 assessment centres. Between 2006 and 2010, approximately 500 000 adults attended a baseline visit and provided written consent. Primary care record linkage was available for a subset, with records starting in 1990 and available until 2017. The completeness of data varied throughout this period, as more primary care practices engaged in electronic coding. In 2019, a follow-up pain questionnaire was conducted online. UK Biobank received ethical approval from the NHS National Research Ethics Service (Ref. 11/NW/0382). This study used application 45465.

Participants self-reporting no recent (<1 month) or chronic pain (3+ months) at baseline were included. Those with a previous diagnosis of primary pain conditions in their primary care record, self-reported fibromyalgia, or serious neurological conditions which may affect cognition or pain reporting were excluded. However, we cannot fully exclude past painful experiences owing to the limitations of available data, and the ubiquity of pain over the course of the lifespan. The analysis of incident primary pain conditions included those with available primary care record data, and the analysis of pain type and severity comprised participants who completed the 2019 pain questionnaire.

### Data collection

At baseline, participants completed questionnaires on lifestyle, family, and social history, and a series of cognitive tasks, using a touchscreen interface. A research nurse collected additional clinical information through face-to-face interviews.

In 2019, participants were invited to complete an online pain questionnaire that comprised the 2016 revised Fibromyalgia Survey Criteria (FSC), which includes the widespread pain index (WPI),^8^ and the Douleur Neuropathique 4 (DN4) interview, a seven-item questionnaire on pain quality.^9^ Although the DN4 is typically used to diagnose neuropathic pain, it may also suggest central sensitisation, a key feature of nociplastic pain.^10^ The WPI was utilised as a measure of nociplastic pain severity.^5^^,^^11^

### Variables

#### CNS symptoms

Three CNS-driven symptoms from the SPACE cluster (sleep, mood, cognitive disturbance) were measured at baseline (we included participants without current pain, and fatigue was not measured at baseline). Each was dichotomised and a count of these features was investigated as an ordinal variable (0–3) to reflect the burden of these symptoms.

Participants were asked ‘Do you have trouble falling asleep at night or do you wake up in the middle of the night?’ with responses ‘never/rarely’, ‘sometimes’, and ‘usually’. Participants were classified as having sleep impairment if they answered ‘usually’, whereas the remaining participants made up the control group.

Participants reporting seeking medical help for mental health problems were classified as having mood disturbance. This broad depression phenotype is a sensitive means of detecting mood disturbance among UK Biobank participants.^12^

Given there was no questionnaire item on self-reported cognitive symptoms, cognitive dysfunction was evaluated using two tests of executive function: reaction time and pairs matching. Executive function performance may correlate with self-reported cognitive symptoms in chronic pain.^13^ A latent variable was derived using confirmatory factor analysis using the ‘lavaan’ package in R, allowing use of a single summary measure. As performance in these tasks covaries, together they may represent a latent variable for executive function.^14^ As there is no agreed cut-off for dysfunction, participants in the lowest quartile were classified as having cognitive dysfunction. Further details are provided in supplement S1.

### Primary pain conditions

The time to diagnosis of a composite outcome of 11 primary pain conditions was assessed using data extracted from linked primary care records via Read v2 and v3 codes mapped to ICD-10 codes (see Supplementary Table S2).^15^ Mapping to ICD-11 codes was not available, as these postdated the primary data in UK Biobank. The conditions comprising the COPCs were selected for analysis, as these share pain characteristics and risk factors, and are common examples of primary pain conditions seen in clinical practice.^15^

### Chronic nociplastic and non-nociplastic pain

Pain status at follow-up was classified into five categories: no pain or nociplastic symptoms (CP– NP–); chronic non-nociplastic pain (CP+ NP–); nociplastic symptoms without pain (CP– NP+); chronic nociplastic pain, further subdivided into possible (CP+ NP+) and probable (CP+ NP++).

Chronic pain (CP+) was defined as pain lasting at least 3 months. Non-nociplastic pain (NP–) was indicated by a DN4 of 0 and FSC <3. Nociplastic pain was classified as possible (NP+; DN4 <3 or FSC 3–11) or probable (NP++; DN4 ≥3^10^ or FSC ≥12^11^). Fibromyalgia criteria require an FSC score ≥12. This classification reflects evidence from prior studies suggesting that many chronic pain conditions—traditionally considered non-neuropathic—can exhibit central sensitisation features. For example, conditions such as fibromyalgia,^16^ osteoarthritis,^17^^,^^18^ and rheumatoid arthritis^19^ have shown associations between high scores on neuropathic pain questionnaires and markers of nociplastic pain. Although the DN4 is primarily validated for neuropathic pain, its inclusion here aims to capture central sensitisation features relevant to nociplastic pain. This approach is supported by literature suggesting that DN4 scores may reflect central sensitisation in non-neuropathic conditions. Nonetheless, we acknowledge the limitation that DN4 is not specifically validated for nociplastic pain classification. To maintain specificity in defining non-nociplastic pain within the CP+ NP– group, only participants with both low DN4 and low FSC scores were included, minimising potential misclassification.

### Nociplastic pain severity

Nociplastic pain severity was measured using the WPI,^11^ a key marker of severity in nociplastic pain conditions,^20^ which may indicate severity in primary pain.

### Covariables

Confounders were selected *a priori* based on evidence of association with both the exposures and primary pain.^20^ Baseline sociodemographic data included age, sex, ethnicity (White, non-White), Townsend index of material deprivation, and education (university degree, no degree). Lifestyle variables included tobacco use (never, former, or current), frequency of alcohol consumption (never, rarely, weekly, daily), and BMI. Age was categorised into 5-yr age bands, and Townsend index into quintiles, for survival analysis, whereas other continuous variables were analysed in their raw form. Field IDs for variables used in this analysis are given in Supplementary Table S1.

### Sample size and missing data

The response options ‘prefer not to answer’ and ‘do not know’ were classified as missing values. A complete case analysis was undertaken as the proportion of missing observations among those who attended the baseline assessment and responded to the 2019 pain questionnaire was <5%.^21^

### Analyses

Descriptive statistics included means and standard deviations (sd) for continuous variables and proportions for categorical variables.

#### Analysis 1: incidence of primary pain conditions

Survival analysis was performed to investigate the relationship between the number of baseline CNS symptoms and time to first diagnosis of a primary pain condition. Given the small proportion of participants with three symptoms, this category was combined with those with two symptoms, leaving three categories: zero, one, two or more symptoms. The first 6 months' follow-up were excluded to reduce the risk of reverse confounding. Participants were censored at the end of their primary care records, date of death, or end of data collection (approximately 2017). A log-rank test was performed to assess for difference in time to diagnosis. Multivariable Cox regression models were fitted for two models, based on hypothesised confounders^20^: unadjusted, and fully adjusted for age, sex, ethnicity, education, Townsend deprivation index, BMI, tobacco, and alcohol use. Owing to non-linearity, age and Townsend deprivation were categorised into five equal quintiles. The proportional hazards assumption was assessed by visual inspection of log-log plots and estimation of Schoenfeld residuals.

#### Analysis 2: chronic nociplastic and non-nociplastic pain at follow-up

Pain status at follow-up was analysed using multinomial logistic regression, with no chronic pain and nociplastic symptoms as the reference group (CP– NP–). Multivariable regression was performed using the same models outlined above. A sensitivity analysis excluding mood, sleep, and cognitive disturbance items from the FSC was also conducted.

#### Analysis 3: nociplastic pain severity

The relationship between baseline symptoms and WPI was assessed using linear regression, using the same multivariable regression models described previously.

The impact of unmeasured confounding on each analysis was examined using E-values. No multicollinearity was found using the variance inflation factor (VIF), with VIF <2 for all included variables) Supplementary Table S3. R version 4.2.0 (R Core Team 2022) was used for all analyses.

## Results

### Study participants

**Fig 1:**
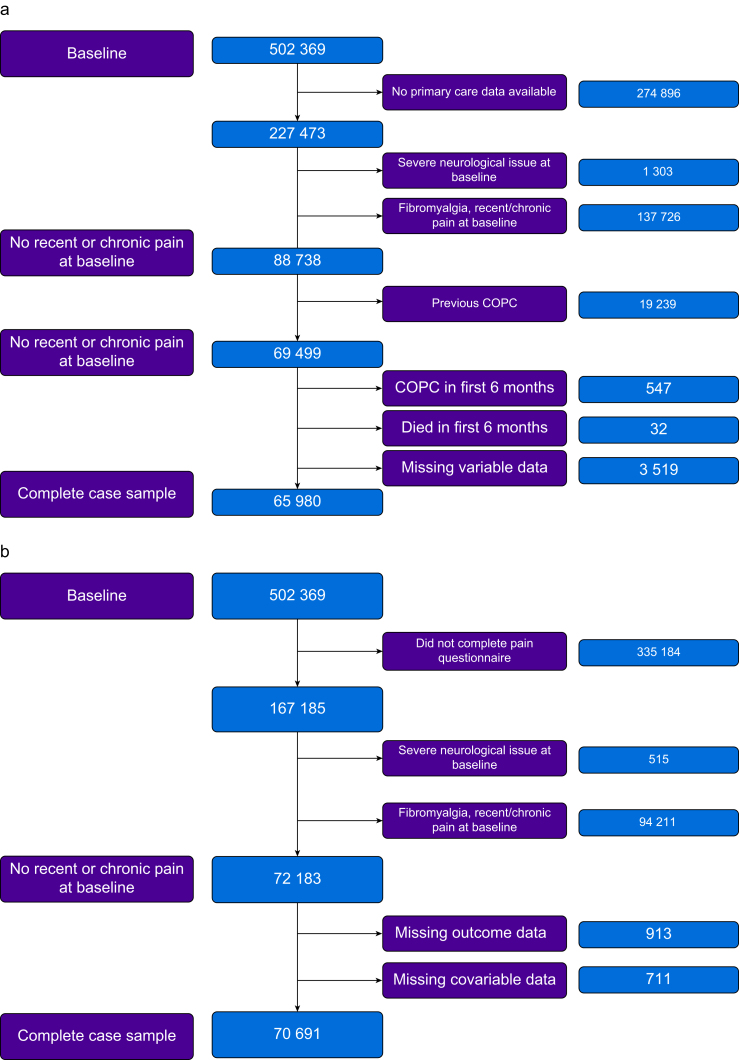
Participant flow diagram for survival analysis with time to diagnosis of complex pain disorders (a), and analysis with follow-up pain questionnaire (b). COPC, chronic overlapping pain syndrome.

Of 502 369 participants at baseline, 227 473 participants had available primary care data, and 65 980 (29.0%) were included in the survival analysis. At follow-up, 167 185 completed the pain questionnaire, and 70 630 (14.0%) were included in the follow-up pain analysis. Most participants were excluded because of unavailability of care records (survival analysis), noncompletion of 2019 pain questionnaire (follow-up analysis), or presence of pain at baseline, with only a small proportion (<5%) missing data owing to incomplete data at baseline or follow-up assessments; see Figure 1 for flow diagram.

### Baseline characteristics

At baseline, 45.9% of participants reported no CNS symptoms, whereas 39.0%, 13.3%, and 1.7% reported one, two, and three symptoms, respectively. Participants with more symptoms exhibited worse executive function, more prevalent sleep issues and mood disturbance. Participants with more symptoms were slightly older, more likely to be female, more deprived, and less likely to be married, employed, or have a university degree. These characteristics displayed a dose–response relationship with the number of symptoms (Table 1). Participants with linked primary care data tended to be slightly older, more likely to be male, White, and more educated, with healthier lifestyle indicators such as lower BMI, less tobacco use, and lower depression/anxiety scores than those without linked primary care data (Supplementary Table S4). The baseline characteristics of participants for the analyses of pain type and severity at follow-up were similar (Supplementary Table S5). Participants who completed the pain questionnaire were slightly younger, more likely to be female, and had higher education levels. They also reported healthier lifestyle factors, such as lower BMI, less tobacco use, and higher cognitive scores than those who did not complete the questionnaire (Supplementary Table S6).

**Table 1:** Baseline characteristics for participants in analysis of time to first chronic primary pain condition. Mean and standard deviation (sd) are reported for continuous variables, and proportions are reported for categorical variables.

		Number of baseline central nervous system symptoms			
	Total	0	1	2	3
	(*N*=65 980)	(*N*=30 321)	(*N*=25 749)	(*N*=8786)	(*N*=1124)
**Age (yr)**					
**Median (range)**	58 (40–70)	56 (40–70)	59 (40–70)	60 (40–70)	61 (40–70)
**Sex**					
**Female, *n* (%)**	33 668 (51)	13 300 (44)	13 996 (54)	5600 (64)	772 (69)
**Male**	32 312 (49)	17 021 (56)	11 753 (46)	3186 (36)	352 (31)
**Ethnicity**					
**Non-White**	2169 (3)	1028 (3)	914 (4)	198 (2)	29 (3)
**White**	63 811 (97)	29 293 (97)	24 835 (96)	8588 (98)	1095 (97)
**Townsend deprivation index**					
**Mean (****sd****)**	–1.62 (2.85)	–1.75 (2.79)	–1.56 (2.87)	–1.43 (2.96)	–1.10 (3.12)
**University degree, *n* (%)**					
**Degree**	24 764 (38)	12 380 (41)	9137 (35)	2928 (33)	319 (28)
**No degree**	41 216 (62)	17 941 (59)	16 612 (65)	5858 (67)	805 (72)
**Tobacco use, *n* (%)**					
**Never**	38 398 (58)	18 649 (62)	14 565 (57)	4592 (52)	592 (53)
**Previous**	21 935 (33)	9350 (31)	8855 (34)	3324 (38)	406 (36)
**Current**	5647 (9)	2322 (8)	2329 (9)	870 (10)	126 (11)
**Alcohol use, *n* (%)**					
**Never**	3971 (6)	1567 (5)	1690 (7)	628 (7)	86 (8)
**Rarely**	12 768 (19)	5463 (18)	5179 (20)	1852 (21)	274 (24)
**Weekly**	34 569 (52)	16 642 (55)	13 163 (51)	4260 (48)	504 (45)
**Daily**	14 672 (22)	6649 (22)	5717 (22)	2046 (23)	260 (23)
**BMI (kg m^−2^)**					
**Mean (****sd****)**	26.8 (4.33)	26.7 (4.21)	26.8 (4.38)	26.8 (4.55)	26.7 (4.64)
**Executive function, Z**					
**Mean (****sd****)**	0.0293 (0.983)	0.480 (0.532)	–0.210 (1.07)	–0.646 (1.12)	–1.36 (0.772)
**Insomnia symptoms, *n* (%)**					
**Never/rarely**	20 187 (31)	12 730 (42)	6562 (25)	895 (10)	0 (0)
**Sometimes**	31 980 (48)	17 591 (58)	12 305 (48)	2084 (24)	0 (0)
**Usually**	13 813 (21)	0 (0)	6882 (27)	5807 (66)	1124 (100)
**Mood disturbance, *n* (%)**					
**No mood disturbance**	48 899 (74)	30 321 (100)	16 237 (63)	2341 (27)	0 (0)
**Mood disturbance**	17 081 (26)	0 (0)	9512 (37)	6445 (73)	1124 (100)
**Number of primary pain conditions during follow-up**					
**Mean (****sd****)**	0.139 (0.380)	0.121 (0.353)	0.146 (0.388)	0.173 (0.428)	0.195 (0.447)
**Follow-up time (days)**					
**Mean (****sd****)**	2710 (768)	2750 (736)	2700 (779)	2630 (824)	2570 (855)

### Analysis 1: incidence of primary pain conditions

**Fig 2:**
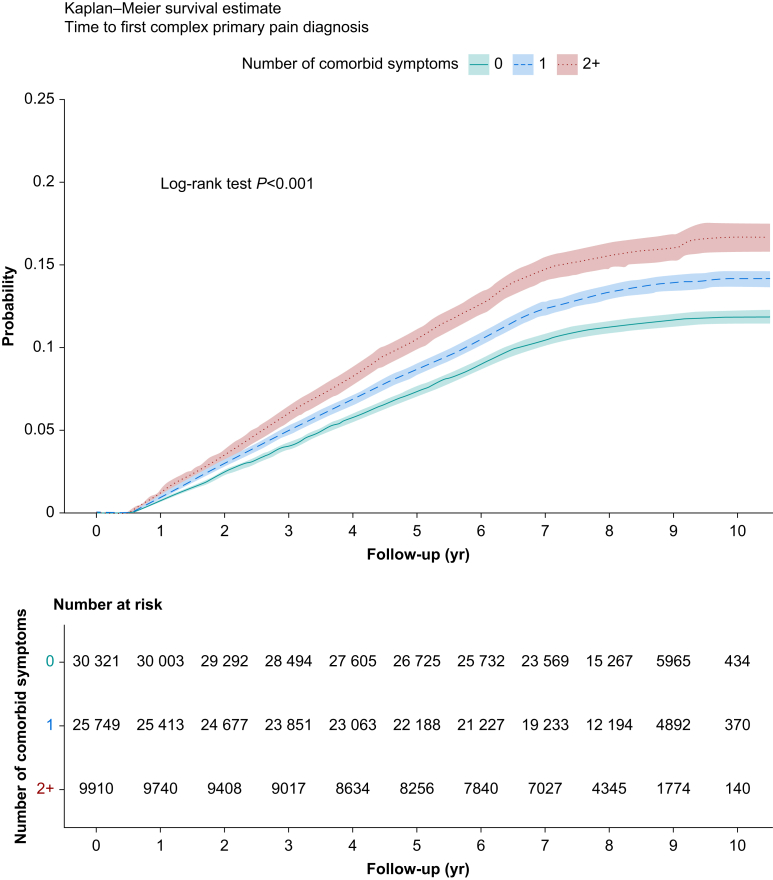
Kaplan–Meier survival curve for time to first diagnosis of a primary pain condition stratified by number of central nervous system-driven symptoms at baseline. The first 6 months of follow-up omitted. Censoring occurred as a result of diagnosis, death, end of primary care record period, or end of data collection.

During a median 8.16 yr of follow-up (range 0.5–11.2 yr) between baseline and diagnosis or the end of GP records, 8086 (12.2%) participants were diagnosed with a primary pain condition. The most common pain diagnosis was low back pain (*n*=5009), accounting for 62% of diagnoses (Supplementary Table S7). Participants with two or more baseline symptoms had a higher risk of developing a primary pain condition (log-rank *P*<0.001; hazard ratio [HR] 1.43, 95% confidence interval [CI] 1.34–1.52; Fig. 2). The association was minimally attenuated after adjusting for confounders in Cox regression (Table 2). In the fully adjusted model, those with two or more baseline symptoms had a 35% greater risk of developing a primary pain condition during follow-up (HR 1.35, 95% CI 1.27–1.44; *P*<0.001) (full results are in Supplementary Table S8). There was no violation of the proportional hazards assumption (Supplementary Figure S1). The E-value for more than two symptoms category in the fully adjusted model was 1.23, indicating that to fully explain away the observed HR of 1.35, an unmeasured confounder would need to be associated with both the exposure and the outcome by a risk ratio of at least 1.23, above and beyond the measured confounders. The E-value for the lower bound of the CI was 1.77, indicating that for the association to be reduced to a nonsignificant level, an unmeasured confounder would need to be associated with both the exposure and the outcome by a risk ratio of at least 1.77.

**Table 2:** Cox proportional hazards regression for association between baseline central nervous system (CNS)-driven symptoms and incidence of chronic primary pain condition. The fully adjusted model included age, sex, ethnicity, education, Townsend deprivation index, BMI, tobacco use, and frequency of alcohol consumption. CI, confidence interval; HR, hazard ratio.

	Unadjusted			Fully adjusted		
	HR	95% CI	*P*	HR	95% CI	*P*
**No. baseline CNS-driven symptoms**						
0						
1	1.2	1.14–1.26	<0.001	1.16	1.10–1.22	<0.001
2+	1.43	1.34–1.52	<0.001	1.35	1.27–1.44	<0.001

### Analysis 2: pain status at follow-up

**Fig 3:**
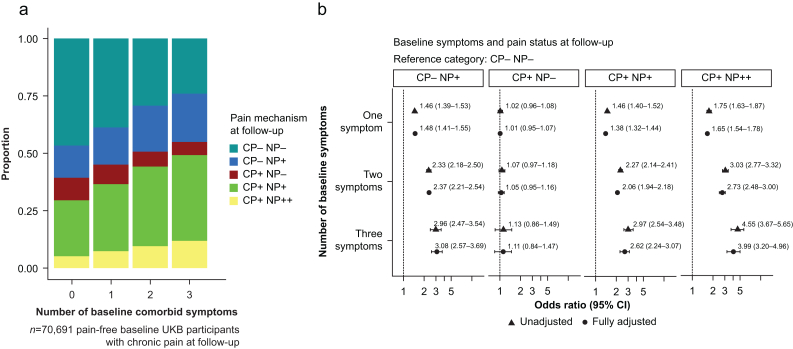
Relationship between count of baseline central nervous system (CNS)-driven symptoms and subsequent pain status after 10 yr of follow-up. (a) Proportion of participants in each pain category at follow-up, according to number of baseline CNS-driven symptoms. (b) Multinomial logistic regression results. The reference category for odds ratios is chronic pain and nociplastic symptom-free individuals (CP– NP–). The count of symptoms displays a positive dose–response relationship with subsequent chronic nociplastic pain, but not chronic non-nociplastic pain. Adjustment for confounding does not markedly attenuate this association. The fully adjusted model included age, sex, ethnicity, education, Townsend deprivation index, BMI, tobacco use, and frequency of alcohol consumption. CI, confidence interval; CP– NP–, chronic non-nociplastic pain; CP+ NP+/++ chronic nociplastic pain; CP– NP+, nociplastic symptoms without chronic pain.

After a median of 10 yr of follow-up (range 8.3–13.6 yr) between baseline and the follow-up pain questionnaire, 30 543 (43.0%) participants reported chronic pain, of whom 24 339 (34.2%) had chronic nociplastic pain (CP+ NP+/++) and 6204 had chronic non-nociplastic pain (8.8%) (CP+ NP–). Participants with more symptoms were more likely to report chronic nociplastic pain (CP+ NP+/++; Fig. 3a). There was a positive dose–response relationship between the number of symptoms at baseline and chronic nociplastic pain (odds ratio [OR] 4.55 for three symptoms; 95% CI 3.67–5.65; *P*<0.001), but not chronic non-nociplastic pain (OR 1.13; 95% CI 0.86–1.49; *P*=0.39) (Fig. 3b). Moreover, the number of baseline symptoms was also associated with greater odds of having nociplastic symptoms but not chronic pain (CP– NP+). Adjusting for age and sex (model 2) slightly attenuated the association, but it remained statistically significant. Further adjustment for sociodemographic and lifestyle factors (fully adjusted model) modestly attenuated the association, but it remained statistically significant (Supplementary Table S9). A sensitivity analysis that omitted the baseline items on mood, sleep, and cognition from the fibromyalgia index did not qualitatively change the results (Supplementary Fig. S2 and Supplementary Table S10). The E-value for the association between three baseline symptoms and CP+/NP++ in the fully adjusted model was 3.99, and the E-value for the lower bound of the CI was 7.44, indicating that for the association to be reduced to a nonsignificant level, an unmeasured confounder would need to be associated with both the exposure and the outcome by a risk ratio of at least 7.44 (Supplementary Table S11).

### Analysis 3: nociplastic pain severity

At follow-up, the mean (sd) widespread index score was 0.86 (1.52). The number of symptoms at baseline was positively associated with more widespread pain at follow-up (Table 3). Unadjusted analyses showed that participants with all three symptoms had a 0.57 (95% CI 0.48–0.67; *P*<0.001) more pain sites at follow-up than those without any baseline symptoms. This association persisted after adjusting for age, sex, sociodemographic, and lifestyle factors (full results in Supplementary Table S12). The E-value for three symptoms in the fully adjusted model was 1.58, and the E-value for the lower bound of the CI was 2.55, indicating that for the association to be reduced to a nonsignificant level, an unmeasured confounder would need to be associated with both the exposure and the outcome by a risk ratio of at least 2.55 (Supplementary Table S13).

**Table 3:** Association between baseline central nervous system (CNS)-driven symptoms and subsequent widespread pain index. The association was investigated using linear regression, and the beta coefficients represent the number of additional pain sites at follow-up compared with those with no baseline CNS symptoms. The count of baseline symptoms displays a positive dose–response relationship with the number of pain sites at follow-up. These associations were not markedly attenuated after adjustment for confounders. The fully adjusted model included age, sex, ethnicity, education, Townsend deprivation index, BMI, tobacco use, and frequency of alcohol consumption. CI, confidence interval.

	Unadjusted			Fully adjusted		
	Beta	95% CI	*P*	Beta	95% CI	*P*
**No. baseline CNS-driven symptoms**						
0						
1	0.191	0.167–0.215	<0.001	0.144	0.119–0.168	<0.001
2	0.434	0.399–0.469	<0.001	0.348	0.313–0.384	<0.001
3	0.574	0.483–0.665	<0.001	0.46	0.370–0.551	<0.001

## Discussion

### Key findings

Our study demonstrates that a greater burden of symptoms from the SPACE cluster is associated with increased risk of developing a primary pain condition in adults with no reported pain at baseline. Furthermore, these symptoms are associated with the presence of chronic nociplastic, but not non-nociplastic, pain after 10 yr of follow-up, and a dose–response relationship with nociplastic pain severity.

### CNS symptoms and pain

Observational studies show a bidirectional link between pain and depression.^22^ In healthy adults, low mood amplified pain unpleasantness via maladaptive thought processes and increased activity in brain regions responsible for pain perception.^23^ This implies that negative mood, such as comorbid depression, may predispose individuals to developing pain. Recent work from UK Biobank also highlights the importance of psychosocial factors, including mood and sleep disturbance, in the spread of pain.^24^

There is a reciprocal relationship between sleep and pain, with sleep disorders possibly causing and resulting from chronic pain.^25^ Experimental studies suggest even a single night of sleep deprivation can cause generalised hyperalgesia,^26^ while insomnia sufferers show increased spontaneous pain and diminished pain inhibition, implying poor sleep quality may contribute to pain development.^27^ Recent work also demonstrates that poor sleep precedes multisite pain onset in children.^28^ Our study identifies poor sleep as a factor predisposing to new-onset nociplastic pain.

Cognitive disturbance is often reported in primary pain conditions (e.g. ‘fibro-fog’ in fibromyalgia) and is commonly viewed as a consequence of pain. However, we demonstrate that worse executive function may predict the development and severity of nociplastic pain. Interestingly, a recent study in children demonstrated attentional problems preceding multisite pain in children,^28^ and a recent ambulatory study of patients with fibromyalgia demonstrated that deterioration in cognitive performance precedes worsening of pain.^29^

Furthermore, we report here that the cumulative burden of these symptoms in a group of adults with no current self-reported pain may predict the later onset of primary pain conditions.

### Underlying mechanisms

Neuroimaging studies in chronic pain show increased functional connectivity between the default mode (DMN), salience (SN), and somatosensory (SMN) networks.^30^ Deficits in the descending pain modulatory system (DPMS) are present in chronic pain conditions, marked by greater pain facilitation and diminished pain inhibition.^31^ The DPMS consists of interconnected cortical, subcortical, and brainstem regions, including the prefrontal cortex (PFC) which is pivotal in the cognitive–affective processing of pain.

An unanswered question is whether these changes are a cause of, or are caused by, pain. Recent work has shown that many of these altered brain connectivity patterns are also present before the development of pain. Children who were initially pain-free but later developed multisite pain displayed increased functional connectivity between regions of the SN, SMN, and DMN, and decreased activity in the medial PFC.^32^ Additionally, cancer patients with increased activity in the periaqueductal grey (PAG), crucial to the DPMS, before treatment were less likely to develop pain after chemotherapy.^33^ These findings imply a pre-existing neural vulnerability to nociplastic pain, although the underlying cause is unknown.

These brain networks also play a major role in sleep, mood, and cognition, potentially linking them to pain vulnerability. Our findings suggest that CNS symptoms may contribute to pain vulnerability, perhaps through alterations in neural networks important for pain perception. However, it is uncertain if these CNS symptoms themselves lead to the development of pain or merely share similar underlying pathology.

### Strengths and limitations

This is the first study to examine predictors of primary pain conditions longitudinally. By also differentiating chronic nociplastic pain from non-nociplastic pain, we show the associations are not solely because of the presence or absence of pain.

Benefiting from a large sample size of participants with no self-reported pain at baseline and a long follow-up period, we attempt to minimise the risk of reverse causation. However, the effect of pain conditions earlier in the life course cannot be excluded. Nevertheless, in a clinical setting, the presence of these symptoms in a currently chronic pain-free adult, should suggest that there is a greater risk of developing a primary chronic pain condition.

Residual confounding may be present; however, adjusting for a comprehensive range of sociodemographic and lifestyle factors had a minimal impact on the observed associations, and sensitivity analysis of E-values suggest moderate robustness to unmeasured confounders. Including only pain-free participants may create a healthy volunteer bias, with our sample being healthier and of a higher socioeconomic position than the general population. Although this might affect external validity, it is unlikely to change the direction of association. There may be gaps in the primary care record data for participants during follow-up, resulting in missed primary pain diagnoses, which may reduce our power to detect an effect. Approximately one-third of participants completed the online pain questionnaire in 2019, with these participants generally reporting better health and sociodemographic characteristics, which may also contribute to a healthy volunteer bias. Similarly, primary care records were only available for 45% of the UKB cohort (see UKB resource 591 v2.0 biobank.ndph.ox.ac.uk/ukb/ukb/docs/primary_care_data.pdf), owing to the nature of the record linkage approach. As these data were missing not at random (MNAR), imputation was not appropriate. However, the incomplete response rate for those who did attend baseline and complete the pain questionnaire was low (<5%), and thus unlikely to introduce bias, leaving a complete case analysis within this group appropriate.^21^

The FSC and the DN4 may not capture all aspects of nociplastic pain, notably clinical signs of pain hypersensitivity. Despite this, the FSC is a reasonable surrogate measure, reflecting key nociplastic pain features.^5^ Although originally developed to identify neuropathic components in pain, tools such as the DN4 and painDETECT also have a role in non-neuropathic conditions where central sensitisation plays a role. For example, studies of patients with osteoarthritis have found that painDETECT scores are correlated with markers of central sensitisation on quantitative sensory testing^17^^,^^34^ and neuroimaging.^18^ Furthermore, in inflammatory arthritis, such as rheumatoid arthritis, neuropathic pain measures can reliably distinguish between pain phenotypes, identifying those whose pain has more centralised characteristics.^19^ In a large population-based study of fibromyalgia, the prototypical nociplastic pain condition, more than half of patients met criteria for ‘neuropathic’ pain,^35^ and patients with fibromyalgia display similar sensory profiles to those with painful diabetic peripheral neuropathy.^16^ Furthermore, in patients with back pain, high painDETECT scores were found to be one of the strongest predictors of fibromyalgia.^36^ Although the painDETECT is more widely studied, it was not available in UK Biobank. However, the DN4 was included in the follow-up pain questionnaire, and this has been shown to be strongly correlated with the painDETECT (rho 0.7, *P*<0.001).^37^ Inclusion of the DN4 thus increases sensitivity to nociplastic pain, which may be identified through high scores on this tool. The association of baseline CNS symptoms with subsequent nociplastic but not non-nociplastic pain emphasises the potential of early CNS symptom assessment in identifying individuals at higher risk for nociplastic conditions, such as fibromyalgia. This differentiation highlights the clinical relevance of CNS symptoms as a predictor of pain types with different management needs.

Although we took measures to ensure specificity by requiring low DN4 and FSC scores in the CP+ NP– group, the overlapping clinical features of nociplastic and neuropathic pain, particularly in conditions such as sciatica, present a potential risk for misclassification that is inherent to screening tools not explicitly designed to differentiate these pain types. Thus, although DN4 was not originally designed for nociplastic pain, it serves here as a surrogate measure owing to its sensitivity to central sensitisation characteristics observed in nociplastic conditions. We acknowledge that some misclassification between nociplastic and neuropathic pain may remain because of the overlap in their clinical features.^38^ The primary distinction between nociplastic and neuropathic pain lies in the presence of a disease or lesion of the somatosensory nervous system, which cannot be directly inferred through current screening questionnaires. Future research should ideally use additional tools such as the Fibromyalgia Rapid Screening Tool **(**FiRST)^39^ or the central sensitisation inventory (CSI),^40^ which are specifically designed to capture nociplastic pain characteristics.

### Clinical implications

This study underlines the importance of early recognition of symptoms such as mood, sleep impairment, and dyscognition, as they may precede, rather than simply result from, primary pain.^3^ Future research should focus on the role of each of these domains on the future development of nociplastic pain. Early treatment of these symptoms, especially in individuals presenting them in combination, could reduce the population burden of primary pain.

### Conclusions

In summary, the cumulative burden of three CNS symptoms identified from the SPACE cluster in a group of middle-aged adults with no self-reported pain predicts the onset of primary pain, and the presence of nociplastic, but not non-nociplastic, pain after a decade. Further mechanistic studies are needed to evaluate if these symptoms are a cause of, or share similar underlying mechanisms as, chronic pain. Exploring early treatments for comorbidities such as depression and insomnia may offer opportunities to decrease future nociplastic pain and reduce its population burden.

## Authors' contributions

Conception and design: all authors

Data collection: EK, AI, IT

Contributed data or analysis tools: EK, AI

Data analysis: EK, AI

Manuscript writing: all authors

Accessed and verified the data: EK, AI

Approved the final version of the manuscript: all authors

## Funding

NIHR Biomedical Research Centre, Oxford; National Institute for Health Research (NIHR) Pfizer Doctoral Fellowship for this research project (NIHR301808 to EK). This paper presents independent research funded by the 10.13039/100018336National Institute for Health Research (NIHR) and Pfizer. The views expressed are those of the author(s) and not necessarily those of the NHS, the NIHR, the Department of Health and Social Care or Pfizer.

## Data sharing

Data is available upon application to UK Biobank. All analysis code will be made publicly available upon publication on an online repository.

## Declaration of interests

EK was supported by a National Institute for Health Research (NIHR) Pfizer Doctoral Fellowship for this research project (NIHR301808). DC and CK are supported by a National Institutes of Health (NIH) grant. AI received funding from UCB, unrelated to the current project, during the time of the project. The other authors declare that they have no conflicts of interest.
