# Supplementary material for: The number of central nervous system-driven symptoms predicts subsequent chronic primary pain: evidence from UK Biobank
Source: Br J Anaesth. 2025 Jan 27;134(3):772–82. doi: 10.1016/j.bja.2024.12.009 (PMC11867065; doi:10.1016/j.bja.2024.12.009)
Supplement: Multimedia component 1 [file mmc1.docx]

| **Variable** | **UKB Datafield** | **Instance** | **Comments** |
| --- | --- | --- | --- |
| Age | 21003 | 0 |  |
| Sex | 31 | 0 |  |
| Ethnicity | 21000 | 0 | Binarised to White and Non-White |
| Townsend Deprivation Index | 22189 | 0 |  |
| Education | 6138 | 0 | Binarised to University Degree and No Degree.  10722 used if 6138 missing. |
| Employment status | 6142 | 0 | Categorised as Employed, Retired, and Unemployed/Other |
| Smoking | 20116 | 0 | Categorised to Current, Previous, and Never smoker |
| Alcohol use | 1558 | 0 | Categorised to Daily, Weekly, Rarely, and Never drinker |
| BMI | 21001 | 0 | 23104 used if 21001 missing |
| Sleep disturbance | 1200 | 0 | Binarised to Usually and Never/Rarely/Sometimes |
| Depression | 2090 & 2100 | 0 | Those who responded “Yes” to either category were categorised as having previous depression |
| Recent Pain | 6159 | 0 | Those who responded “None of the above” were categorised as having no pain at baseline |
| Chronic pain variables |  | 0 | 3799 Headaches for 3+ months  4067 Facial pains for 3+ months  3404 Neck/shoulder pain for 3+ months  3571 Back pain for 3+ months  3741 Stomach/abdominal pain for 3+ months  3414 Hip pain for 3+ months  3773 Knee pain for 3+ months  2956 General pain for 3+ months |
| Exclusion criteria | 20002 | 0 | Dementia: 1263  Parkinson’s disease: 1262  Psychosis: 1289  Bipolar disorder: 1291 |
| Chronic Overlapping Pain Conditions | 20002 | 0 | Fibromyalgia: 1542  IBS: 1154  Cystitis: 1514  Prostatitis: 1517  Migraine: 1265  Headache: 1436  Back pain: 1294 & 1534  CFS: 1482  Endometriosis: 1402 |
| Reaction time | 20023 | 0 | Winsorised: Values >1200 msec and <300 msec were replaced with 1200 and 300 respectively |
| Pairs matching | 399 | 0 | Round 2 used  10137 used if 399 missing  Values >22 were replaced with 2 |
| Responded to Experience of Pain Questionnaire | 120128 | PQ | Missing values for the questionnaire date were taken as non-response |
| Chronic pain on pain questionnaire | 120019 | PQ |  |
| DN4 Questionnaire | 120046  120047  120048  120049  120050  120051  120052 | PQ | Those who reported no pain on 120019 were coded as not having a DN4 score of 0 |
| Widespread Pain Index | 120039 | PQ | Sum of the number of pain areas.  Those who reported “Undisclosed area” were regarded as missing |
| Symptom Severity Scale | 120041  120042  120043  120044  120045 | PQ | Sum of these scores.  Those with missing responses to any item were regarded as missing |
| Follow-up time from baseline to pain questionnaire | Baseline date: 53  Questionnaire date: 120128 | 0 & PQ | The difference between these two dates |
| Survival Analysis: |  |  |  |
| Presence of Primary Care Records | 42040 | 0 | Only participants with primary care records were included |
| Date of death | 40000 | 0 |  |

Supplementary Table S1. UK Biobank data fields used in analysis. Note Item responses “prefer not to answer” and “do not know” were coded as missing. Participants who withdrew from UK Biobank as of 25/04/2023 were removed from the dataset. Instance “0” refers to the baseline 2006-2010 visit. “PQ” refers to the 2019 pain questionnaire.

Supplement S1. CNS symptoms.

Latent variable for cognition:

- Cognitive test variables were winsorised to remove outliers as follows:
- Reaction time >1200msec (99^th^ percentile) or <300msec (1^st^ percentile) were winsorised and imputed with 1200msec and 300msec respectively.
- Incorrect pair matches >22 (99^th^ percentile) were winsorised and imputed with 22.
- Cognitive test variables were re-scaled using the proportion of maximum score method (Little T, 2013) so that all cognitive task variables were on a scale of 0 to 1, where higher scores indicated worse cognition.
- Using the *Lavaan* package in R, a one factor confirmatory factor analysis model was used to construct a latent variable for cognition.
- Full information maximum likelihood estimation was used to account for missing observations.
- Robust estimation was used (“MLR” option).
- A RMSEA <0.05, and CFI & TLI >0.9 was used to indicate acceptable model fit.
- These were met, with CFI and TLI both = 1.0, and RMSEA <0.05.
- The latent variable for cognition was extracted. This was then centred on a mean of 0 and standardised.
- Those in the bottom quartile were categorised as poor cognition for the analyses.

Baseline symptoms:

- Those who responded “Usually” to self-reported sleep difficulties were categorised as having sleep disturbance.
- Those who responded “Yes” to seeking help for mood or nerve problems with a GP or psychiatrist were categorised as having mood disturbance.
- Those in the bottom quartile of cognition were categorised as having cognitive disturbance.
- The sum of these variables was calculated to give an ordinal variable from 0 (no symptoms) to 3 (all three symptoms).
- Due to a small number of participants with three symptoms in the survival analysis, this was combined with those with two symptoms to give three categories: 0, 1 ,2+ symptoms.

| **Chronic primary pain condition** | **ICD-10 code** | **Read v2** | **ICD10** | **Read v3** | **ICD10** | **Read v3 Mapping Status** | **Number in UKB** |  |  |
| --- | --- | --- | --- | --- | --- | --- | --- | --- | --- |
| Fibromyalgia | M79.7 | N239. | M797 | N240. | M797 | D | 2497 |  |  |
| Fibromyalgia |  | N2401 | M797 | N2401 | M797 | G |  |  |  |
| Fibromyalgia |  | N2405 | M797 | N2405 | M797 | G |  |  |  |
| Fibromyalgia |  | N2406 | M797 | N2406 | M797 | G |  |  |  |
| Fibromyalgia |  | N2412 | M797 | N240z | M797 | D |  |  |  |
| Fibromyalgia |  | N248. | M797 | X708C | M797 | D |  |  |  |
| Fibromyalgia |  | N2480 | M797 | X75rx | M797 | G |  |  |  |
| Irritable Bowel Syndrome | K58.0 | J5210 | K580 | J5210 | K580 | D | 18342 |  |  |
| Irritable Bowel Syndrome | K58.1 | J5212 | K580 | X3060 | K580 | G |  |  |  |
| Irritable Bowel Syndrome | K58.2 | J521. | K589 | XabiQ | K580 | G |  |  |  |
| Irritable Bowel Syndrome | K58.8 | J5211 | K589 | XE0as | K580 | A |  |  |  |
| Irritable Bowel Syndrome | K58.9 |  |  | XM0z0 | K580 | R |  |  |  |
| Irritable Bowel Syndrome |  |  |  | J4... | K589 | G |  |  |  |
| Irritable Bowel Syndrome |  |  |  | J521. | K589 | G |  |  |  |
| Irritable Bowel Syndrome |  |  |  | X305z | K589 | G |  |  |  |
| Irritable Bowel Syndrome |  |  |  | X3061 | K589 | G |  |  |  |
| Irritable Bowel Syndrome |  |  |  | X76dS | K589 | G |  |  |  |
| Irritable Bowel Syndrome |  |  |  | XabiP | K589 | G |  |  |  |
| Irritable Bowel Syndrome |  |  |  | XE0as | K589 | D |  |  |  |
| Irritable Bowel Syndrome |  |  |  | XM0z0 | K589 | R |  |  |  |
| Interstitial Cystitis | N30.10 | K151. | N301 | K151. | N301 | G | 288 |  |  |
| Interstitial Cystitis | N30.30 | K1510 | N301 | K1510 | N301 | D |  |  |  |
| Interstitial Cystitis |  | K1511 | N301 | K1511 | N301 | G |  |  |  |
| Interstitial Cystitis |  | K1512 | N301 | K1512 | N301 | G |  |  |  |
| Interstitial Cystitis |  | K151z | N301 | K151z | N301 | D |  |  |  |
| Interstitial Cystitis |  | K153. | N303 | X30Nb | N301 | A |  |  |  |
| Interstitial Cystitis |  | K1530 | N303 | X30Nc | N301 | A |  |  |  |
| Interstitial Cystitis |  | K1531 | N303 | X30NX | N301 | A |  |  |  |
| Interstitial Cystitis |  | K1532 | N303 | Xa8EK | N301 | A |  |  |  |
| Interstitial Cystitis |  | K153z | N303 | K153. | N303 | G |  |  |  |
| Interstitial Cystitis |  |  |  | K1530 | N303 | G |  |  |  |
| Interstitial Cystitis |  |  |  | K1531 | N303 | G |  |  |  |
| Interstitial Cystitis |  |  |  | K1532 | N303 | G |  |  |  |
| Interstitial Cystitis |  |  |  | K153z | N303 | D |  |  |  |
| Interstitial Cystitis |  |  |  | XE0dq | N303 | E |  |  |  |
| Chronic Prostatitis | N41.1 | K211. | N411 | K211. | N411 | E | 420 |  |  |
| Vulvodynia | N94.810 | K42y. | N948 | 2689. | N948 | G | 1314 |  |  |
| Vulvodynia | N94.818 | K585. | N948 | 1A58. | N948 | D |  |  |  |
| Vulvodynia | N94.819 | K58y. | N948 | 1A581 | N948 | G |  |  |  |
| Vulvodynia |  | K58y0 | N948 | 1AE.. | N948 | G |  |  |  |
| Vulvodynia |  | K59y0 | N948 | 1C6Z. | N948 | R |  |  |  |
| Vulvodynia |  | K59y1 | N948 | K23.. | N948 | R |  |  |  |
| Vulvodynia |  | K59y2 | N948 | K585. | N948 | G |  |  |  |
| Vulvodynia |  | K5C.. | N948 | K58y. | N948 | D |  |  |  |
| Vulvodynia |  | K5C0. | N948 | K59y0 | N948 | G |  |  |  |
| Vulvodynia |  | K5C1. | N948 | K59y1 | N948 | D |  |  |  |
| Vulvodynia |  | K5Cy. | N948 | K59y2 | N948 | D |  |  |  |
| Vulvodynia |  | K5Cz. | N948 | K5C0. | N948 | D |  |  |  |
| Vulvodynia |  | K5y.. | N948 | K5C1. | N948 | G |  |  |  |
| Vulvodynia |  | Kyu9E | N948 | K5Cy. | N948 | D |  |  |  |
| Vulvodynia |  |  |  | K5Cz. | N948 | G |  |  |  |
| Vulvodynia |  |  |  | K5y.. | N948 | D |  |  |  |
| Vulvodynia |  |  |  | Kyu9E | N948 | E |  |  |  |
| Vulvodynia |  |  |  | N2472 | N948 | R |  |  |  |
| Vulvodynia |  |  |  | X309a | N948 | R |  |  |  |
| Vulvodynia |  |  |  | X30Of | N948 | G |  |  |  |
| Vulvodynia |  |  |  | X400l | N948 | R |  |  |  |
| Vulvodynia |  |  |  | X76QB | N948 | G |  |  |  |
| Vulvodynia |  |  |  | X76QD | N948 | G |  |  |  |
| Vulvodynia |  |  |  | X76QM | N948 | D |  |  |  |
| Vulvodynia |  |  |  | X76QS | N948 | G |  |  |  |
| Vulvodynia |  |  |  | X78Rg | N948 | R |  |  |  |
| Vulvodynia |  |  |  | X78Rn | N948 | R |  |  |  |
| Vulvodynia |  |  |  | Xa0bk | N948 | G |  |  |  |
| Vulvodynia |  |  |  | Xa0l8 | N948 | R |  |  |  |
| Vulvodynia |  |  |  | Xa0wh | N948 | G |  |  |  |
| Vulvodynia |  |  |  | Xa0wi | N948 | G |  |  |  |
| Vulvodynia |  |  |  | Xa2XA | N948 | D |  |  |  |
| Vulvodynia |  |  |  | Xa2XF | N948 | D |  |  |  |
| Vulvodynia |  |  |  | Xa2XI | N948 | D |  |  |  |
| Vulvodynia |  |  |  | Xa4aF | N948 | D |  |  |  |
| Vulvodynia |  |  |  | Xa4aG | N948 | D |  |  |  |
| Vulvodynia |  |  |  | Xa4cl | N948 | G |  |  |  |
| Vulvodynia |  |  |  | Xa4cn | N948 | G |  |  |  |
| Vulvodynia |  |  |  | Xa4dA | N948 | G |  |  |  |
| Vulvodynia |  |  |  | Xa4dC | N948 | G |  |  |  |
| Vulvodynia |  |  |  | Xa4dD | N948 | G |  |  |  |
| Vulvodynia |  |  |  | Xa4Z2 | N948 | G |  |  |  |
| Vulvodynia |  |  |  | Xa6nx | N948 | G |  |  |  |
| Vulvodynia |  |  |  | Xa6YA | N948 | G |  |  |  |
| Vulvodynia |  |  |  | Xa7HI | N948 | D |  |  |  |
| Vulvodynia |  |  |  | Xa7HJ | N948 | G |  |  |  |
| Vulvodynia |  |  |  | Xa7js | N948 | G |  |  |  |
| Vulvodynia |  |  |  | Xa89q | N948 | G |  |  |  |
| Vulvodynia |  |  |  | Xa96W | N948 | R |  |  |  |
| Vulvodynia |  |  |  | XaIP9 | N948 | R |  |  |  |
| Vulvodynia |  |  |  | XaJhL | N948 | G |  |  |  |
| Vulvodynia |  |  |  | XC0eg | N948 | D |  |  |  |
| Vulvodynia |  |  |  | XE0ez | N948 | D |  |  |  |
| Vulvodynia |  |  |  | XM0z0 | N948 | R |  |  |  |
| Vulvodynia |  |  |  | XM0zT | N948 | R |  |  |  |
| Migraine | G43.XXX | F261. | G430 | 1BA4. | G43 | R | 17,825 |  |  |
| Migraine | Excluding G43.6 | F261z | G430 | 1BB1. | G43 | R |  |  |  |
| Migraine | Excluding G43.A | F260. | G431 | 1BB4. | G43 | R |  |  |  |
| Migraine |  | F2623 | G431 | F26.. | G43 | A |  |  |  |
| Migraine |  | F26y0 | G431 | XaXkr | G43 | A |  |  |  |
| Migraine |  | R090D | G431 | XE0rh | G43 | R |  |  |  |
| Migraine |  | F26y2 | G432 | XE2rs | G43 | R |  |  |  |
| Migraine |  | F26y3 | G433 | F261. | G430 | G |  |  |  |
| Migraine |  | F2610 | G438 | F261z | G430 | D |  |  |  |
| Migraine |  | F262. | G438 | F260. | G431 | G |  |  |  |
| Migraine |  | F2621 | G438 | F2623 | G431 | G |  |  |  |
| Migraine |  | F2622 | G438 | F26y0 | G431 | G |  |  |  |
| Migraine |  | F2624 | G438 | X007J | G431 | G |  |  |  |
| Migraine |  | F262z | G438 | X007K | G431 | G |  |  |  |
| Migraine |  | F26y. | G438 | X007L | G431 | G |  |  |  |
| Migraine |  | F26y1 | G438 | X007N | G431 | G |  |  |  |
| Migraine |  | F26yz | G438 | X007R | G432 | E |  |  |  |
| Migraine |  | Fyu53 | G438 | F26y3 | G433 | D |  |  |  |
| Migraine |  | F26.. | G439 | X007S | G433 | G |  |  |  |
| Migraine |  | F26z. | G439 | 1967. | G438 | G |  |  |  |
| Migraine |  |  |  | F2610 | G438 | D |  |  |  |
| Migraine |  |  |  | F262. | G438 | D |  |  |  |
| Migraine |  |  |  | F2622 | G438 | G |  |  |  |
| Migraine |  |  |  | F2624 | G438 | D |  |  |  |
| Migraine |  |  |  | F262z | G438 | D |  |  |  |
| Migraine |  |  |  | F26y. | G438 | D |  |  |  |
| Migraine |  |  |  | F26y1 | G438 | G |  |  |  |
| Migraine |  |  |  | F26yz | G438 | D |  |  |  |
| Migraine |  |  |  | Fyu53 | G438 | E |  |  |  |
| Migraine |  |  |  | R090D | G438 | G |  |  |  |
| Migraine |  |  |  | X007M | G438 | G |  |  |  |
| Migraine |  |  |  | X007O | G438 | G |  |  |  |
| Migraine |  |  |  | F26.. | G439 | D |  |  |  |
| Migraine |  |  |  | F26z. | G439 | D |  |  |  |
| Migraine |  |  |  | XaXkr | G439 | D |  |  |  |
| Chronic tension-type headache | G44.201 | F2626 | G442 | X007U | G442 | G | 3931 |  |  |
| Chronic tension-type headache | G44.209 | F2629 | G442 | X007V | G442 | G |  |  |  |
| Chronic tension-type headache | G44.211 | F262A | G442 | Xa0wU | G442 | A |  |  |  |
| Chronic tension-type headache | G44.219 | F262B | G442 | XaXpZ | G442 | G |  |  |  |
| Chronic tension-type headache | G44.221 |  |  | XaXsF | G442 | G |  |  |  |
| Chronic tension-type headache | G44.229 |  |  | XE1Yl | G442 | E |  |  |  |
| Temporomandibular Disorder | M26.60 | S40.. | S030 | J046. | S030 | R | 2200 |  |  |
| Temporomandibular Disorder | M26.62 | S400. | S030 | S40.. | S030 | G | 72 |  |  |
| Temporomandibular Disorder | M.26.63 | S401. | S030 | S400. | S030 | D |  |  |  |
| Temporomandibular Disorder | S03.0XXA | S402. | S030 | S401. | S030 | D |  |  |  |
| Temporomandibular Disorder | | S403. | S030 | S402. | S030 | D | | |  |
| Temporomandibular Disorder | | S40z. | S030 | S403. | S030 | D | | |  |
| Temporomandibular Disorder | |  |  | S40z. | S030 | D | | |  |
| Temporomandibular Disorder | |  |  | XA0Ce | S030 | G | | |  |
| Temporomandibular Disorder | |  |  | XA0DI | S030 | G | | |  |
| Temporomandibular Disorder | |  |  | Xa9DB | S030 | G | | |  |
| Temporomandibular Disorder | |  |  | XE1lK | S030 | G | | |  |
| Chronic Low Back Pain | M54.5 | N1420 | M544 | 16C.. | M545 | A | 61,294 |  |  |
| Chronic Low Back Pain | M54.40 | N142. | M545 | 25E5. | M545 | G |  |  |  |
| Chronic Low Back Pain | M54.41 | R090C | M545 | 25E7. | M545 | G |  |  |  |
| Chronic Low Back Pain | M54.41 | N13y2 | M548 | N142. | M545 | G |  |  |  |
| Chronic Low Back Pain | M54.89 | N14A. | M548 | R090C | M545 | D |  |  |  |
| Chronic Low Back Pain |  | N14y. | M548 | X30Px | M545 | D |  |  |  |
| Chronic Low Back Pain |  | Nyu7A | M548 | X75s1 | M545 | G |  |  |  |
| Chronic Low Back Pain |  |  |  | X75s3 | M545 | G |  |  |  |
| Chronic Low Back Pain |  |  |  | Xa0sK | M545 | G |  |  |  |
| Chronic Low Back Pain |  |  |  | Xa0sM | M545 | G |  |  |  |
| Chronic Low Back Pain |  |  |  | Xa0wt | M545 | D |  |  |  |
| Chronic Low Back Pain |  |  |  | Xa0wu | M545 | G |  |  |  |
| Chronic Low Back Pain |  |  |  | Xa0yD | M545 | G |  |  |  |
| Chronic Low Back Pain |  |  |  | Xa70A | M545 | D |  |  |  |
| Chronic Low Back Pain |  |  |  | Xa7mA | M545 | G |  |  |  |
| Chronic Low Back Pain |  |  |  | Xa7xj | M545 | G |  |  |  |
| Chronic Low Back Pain |  |  |  | Xa7xk | M545 | G |  |  |  |
| Chronic Low Back Pain |  |  |  | XE1FB | M545 | D |  |  |  |
| Chronic Low Back Pain |  |  |  | XM092 | M545 | G |  |  |  |
| Chronic Low Back Pain |  |  |  | XM0Cp | M545 | G |  |  |  |
| Chronic Low Back Pain |  |  |  | XM0Cq | M545 | G |  |  |  |
| Chronic Low Back Pain |  |  |  | XM1GI | M545 | A |  |  |  |
| Chronic Low Back Pain |  |  |  | XM1NI | M545 | G |  |  |  |
| Chronic Low Back Pain |  |  |  | N1420 | M544 | D |  |  |  |
| Chronic Low Back Pain |  |  |  | X75rz | M544 | D |  |  |  |
| Chronic Low Back Pain |  |  |  | X75sm | M544 | R |  |  |  |
| Chronic Low Back Pain |  |  |  | Xa70A | M544 | A |  |  |  |
| Chronic Low Back Pain |  |  |  | XaB0c | M544 | A |  |  |  |
| Chronic Low Back Pain |  |  |  | XaB0d | M544 | A |  |  |  |
| Chronic Low Back Pain |  |  |  | XE1FB | M544 | A |  |  |  |
| Chronic Low Back Pain |  |  |  | XE1FC | M544 | A |  |  |  |
| Chronic Fatigue Syndrome | R53.82 | R007. | R53X | 1682. | R53X | D | 22554 |  |  |
| Chronic Fatigue Syndrome | | R0070 | R53X | R007. | R53X | D | | |  |
| Chronic Fatigue Syndrome | | R0071 | R53X | R0070 | R53X | D | | |  |
| Chronic Fatigue Syndrome | | R0072 | R53X | R0071 | R53X | D | | |  |
| Chronic Fatigue Syndrome | | R0073 | R53X | R0072 | R53X | D | | |  |
| Chronic Fatigue Syndrome | | R0074 | R53X | R0073 | R53X | D | | |  |
| Chronic Fatigue Syndrome | | R0075 | R53X | R0074 | R53X | D | | |  |
| Chronic Fatigue Syndrome | | R007z | R53X | R0075 | R53X | D | | |  |
| Chronic Fatigue Syndrome | | R2y3. | R53X | R007z | R53X | D | | |  |
| Chronic Fatigue Syndrome | |  |  | Ua150 | R53X | G | | |  |
| Chronic Fatigue Syndrome | |  |  | X0081 | R53X | G | | |  |
| Chronic Fatigue Syndrome | |  |  | X70xR | R53X | G | | |  |
| Chronic Fatigue Syndrome | |  |  | X76Ad | R53X | G | | |  |
| Chronic Fatigue Syndrome | |  |  | X76Ae | R53X | G | | |  |
| Chronic Fatigue Syndrome | |  |  | X76Af | R53X | D | | |  |
| Chronic Fatigue Syndrome | |  |  | X76Ag | R53X | D | | |  |
| Chronic Fatigue Syndrome | |  |  | X76Ai | R53X | G | | |  |
| Chronic Fatigue Syndrome | |  |  | X76Aj | R53X | G | | |  |
| Chronic Fatigue Syndrome | |  |  | X76Am | R53X | D | | |  |
| Chronic Fatigue Syndrome | |  |  | X76An | R53X | D | | |  |
| Chronic Fatigue Syndrome | |  |  | X76Ao | R53X | D | | |  |
| Chronic Fatigue Syndrome | |  |  | Xa35q | R53X | G | | |  |
| Chronic Fatigue Syndrome | |  |  | Xa96S | R53X | D | | |  |
| Chronic Fatigue Syndrome | |  |  | Xabdb | R53X | D | | |  |
| Chronic Fatigue Syndrome | |  |  | Xabdd | R53X | D | | |  |
| Chronic Fatigue Syndrome | |  |  | XabdY | R53X | D | | |  |
| Chronic Fatigue Syndrome | |  |  | XaEXl | R53X | G | | |  |
| Chronic Fatigue Syndrome | |  |  | XE0qk | R53X | G | | |  |
| Chronic Fatigue Syndrome | |  |  | XE0ql | R53X | G | | |  |
| Chronic Fatigue Syndrome | |  |  | XE0UW | R53X | D | | |  |
| Chronic Fatigue Syndrome | |  |  | XE2y5 | R53X | D | | |  |
| Chronic Fatigue Syndrome | |  |  | XM06l | R53X | G | | |  |
| Chronic Fatigue Syndrome | |  |  | XM06o | R53X | G | | |  |
| Chronic Fatigue Syndrome | |  |  | XM0Ce | R53X | G | | |  |
| Chronic Fatigue Syndrome | |  |  | XM0D3 | R53X | D | | |  |
| Chronic Fatigue Syndrome | |  |  | XM0D5 | R53X | G | | |  |
| Chronic Fatigue Syndrome | |  |  | XM0yx | R53X | D | | |  |
| Chronic Fatigue Syndrome | |  |  | XM1AV | R53X | D | | |  |
| Endometriosis | N80.XXX | K500. | N800 | X408N | N80 | A | 2611 |  |  |
| Endometriosis |  | K5000 | N800 | XE0eW | N80 | A |  |  |  |
| Endometriosis |  | K5001 | N800 | K500. | N800 | G |  |  |  |
| Endometriosis |  | K500z | N800 | K5000 | N800 | D |  |  |  |
| Endometriosis |  | K50.. | N800-N809 | K500z | N800 | D |  |  |  |
| Endometriosis |  | K501. | N801 | X408O | N800 | G |  |  |  |
| Endometriosis |  | K502. | N802 | K501. | N801 | E |  |  |  |
| Endometriosis |  | K503. | N803 | X408R | N801 | G |  |  |  |
| Endometriosis |  | K5030 | N803 | XE0eX | N801 | E |  |  |  |
| Endometriosis |  | K5031 | N803 | K502. | N802 | E |  |  |  |
| Endometriosis |  | K5032 | N803 | K503. | N803 | G |  |  |  |
| Endometriosis |  | K5033 | N803 | K5030 | N803 | G |  |  |  |
| Endometriosis |  | K503z | N803 | K5031 | N803 | G |  |  |  |
| Endometriosis |  | K504. | N804 | K5032 | N803 | G |  |  |  |
| Endometriosis |  | K5040 | N804 | K5033 | N803 | G |  |  |  |
| Endometriosis |  | K5041 | N804 | K503z | N803 | D |  |  |  |
| Endometriosis |  | K504z | N804 | X408N | N803 | D |  |  |  |
| Endometriosis |  | K505. | N805 | XaEWA | N803 | G |  |  |  |
| Endometriosis |  | K5050 | N805 | K504. | N804 | G |  |  |  |
| Endometriosis |  | K5051 | N805 | K5040 | N804 | G |  |  |  |
| Endometriosis |  | K5052 | N805 | K5041 | N804 | G |  |  |  |
| Endometriosis |  | K505z | N805 | K504z | N804 | D |  |  |  |
| Endometriosis |  | K506. | N806 | K505. | N805 | E |  |  |  |
| Endometriosis |  | K5002 | N808 | K5050 | N805 | G |  |  |  |
| Endometriosis |  | K50y. | N808 | K5051 | N805 | G |  |  |  |
| Endometriosis |  | K50y0 | N808 | K5052 | N805 | G |  |  |  |
| Endometriosis |  | K50y1 | N808 | K505z | N805 | D |  |  |  |
| Endometriosis |  | K50y2 | N808 | K506. | N806 | G |  |  |  |
| Endometriosis |  | K50y3 | N808 | X408T | N806 | A |  |  |  |
| Endometriosis |  | K50yz | N808 | X408V | N806 | G |  |  |  |
| Endometriosis |  | Kyu90 | N808 | K5002 | N808 | G |  |  |  |
| Endometriosis |  | K50z. | N809 | K50y. | N808 | D |  |  |  |
| Endometriosis |  |  |  | K50y0 | N808 | G |  |  |  |
| Endometriosis |  |  |  | K50y1 | N808 | G |  |  |  |
| Endometriosis |  |  |  | K50y2 | N808 | G |  |  |  |
| Endometriosis |  |  |  | K50y3 | N808 | G |  |  |  |
| Endometriosis |  |  |  | K50yz | N808 | D |  |  |  |
| Endometriosis | AND | R090G | R102 | K50z. | N808 | D |  |  |  |
| Endometriosis | R10.2 | K5830 | N944 | Kyu90 | N808 | E |  |  |  |
| Endometriosis | N94.4 / 5 / 6 | K5831 | N945 | X101N | N808 | G |  |  |  |
| Endometriosis | N94.10 / 11 / 12/ 19 | K583. | N946 | X408T | N808 | D |  |  |  |
| Endometriosis |  | K580. | N941 | XE0eW | N809 | D |  |  |  |
| Endometriosis |  |  |  | 197C. | R102 | A |  |  |  |
| Endometriosis |  |  |  | 1A5.. | R102 | G |  |  |  |
| Endometriosis |  |  |  | 25C8. | R102 | G |  |  |  |
| Endometriosis |  |  |  | R090G | R102 | D |  |  |  |
| Endometriosis |  |  |  | Ua1eG | R102 | G |  |  |  |
| Endometriosis |  |  |  | X4072 | R102 | G |  |  |  |
| Endometriosis |  |  |  | X50GC | R102 | G |  |  |  |
| Endometriosis |  |  |  | X75rf | R102 | G |  |  |  |
| Endometriosis |  |  |  | Xa0wm | R102 | G |  |  |  |
| Endometriosis |  |  |  | Xa84T | R102 | A |  |  |  |
| Endometriosis |  |  |  | Xa84t | R102 | G |  |  |  |
| Endometriosis |  |  |  | XaFEC | R102 | G |  |  |  |
| Endometriosis |  |  |  | XM00l | R102 | G |  |  |  |
| Endometriosis |  |  |  | XM118 | R102 | A |  |  |  |
| Endometriosis |  |  |  | XM1BA | R102 | E |  |  |  |
| Endometriosis |  |  |  | X408t | N944 | E |  |  |  |
| Endometriosis |  |  |  | Xa3g8 | N944 | A |  |  |  |
| Endometriosis |  |  |  | XE0ek | N944 | A |  |  |  |
| Endometriosis |  |  |  | X408u | N945 | E |  |  |  |
| Endometriosis |  |  |  | Xa3g8 | N945 | A |  |  |  |
| Endometriosis |  |  |  | XE0ek | N945 | A |  |  |  |
| Endometriosis |  |  |  | K583. | N946 | G |  |  |  |
| Endometriosis |  |  |  | N2472 | N946 | R |  |  |  |
| Endometriosis |  |  |  | X408s | N946 | G |  |  |  |
| Endometriosis |  |  |  | Xa3g8 | N946 | D |  |  |  |
| Endometriosis |  |  |  | Xa4e8 | N946 | G |  |  |  |
| Endometriosis |  |  |  | XE0ek | N946 | D |  |  |  |
| Endometriosis |  |  |  | XM0zT | N946 | R |  |  |  |
| Endometriosis |  |  |  | K580. | N941 | D |  |  |  |
| Endometriosis |  |  |  | X75ta | N941 | G |  |  |  |
| Endometriosis |  |  |  | X75tU | N941 | D |  |  |  |
| Endometriosis |  |  |  | X75tV | N941 | D |  |  |  |
| Endometriosis |  |  |  | X75tW | N941 | D |  |  |  |
| Endometriosis |  |  |  | X75tX | N941 | D |  |  |  |
| Endometriosis |  |  |  | Xa0y2 | N941 | G |  |  |  |
| Endometriosis |  |  |  | Xa3g7 | N941 | D |  |  |  |
| Endometriosis |  |  |  | XaEJW | N941 | D |  |  |  |
|  |  |  |  |  |  |  |  |  |  |
| Osteoarthritis |  | N0504 | M15.0 | N050. | M15 | A | 36,360 |  |  |
|  |  | N0507 | M15.1 | N05zE | M15 | R |  |  |  |
|  |  | N0503 | M15.2 | X703B | M15 | R |  |  |  |
|  |  | N0505 | M15.3 | X703C | M15 | R |  |  |  |
|  |  | N0506 | M15.4 | X703D | M15 | R |  |  |  |
|  |  | Nyu20 | M15.8 | X703E | M15 | R |  |  |  |
|  |  | N050. | M15.9 | X703F | M15 | R |  |  |  |
|  |  | N0500 | M16.0 | X703G | M15 | R |  |  |  |
|  |  | N0501 | M16.1 | X703H | M15 | R |  |  |  |
|  |  | N0502 | M16.2 | X703I | M15 | R |  |  |  |
|  |  | N050z | M16.3 | X703K | M15 | R |  |  |  |
|  |  | N0519 | M16.4 | X703L | M15 | R |  |  |  |
|  |  | N0515 | M16.5 | X703M | M15 | R |  |  |  |
|  |  | Nyu21 | M16.6 | X703N | M15 | R |  |  |  |
|  |  | N051A | M16.7 | X703O | M15 | R |  |  |  |
|  |  | Nyu22 | M16.8 | X703P | M15 | R |  |  |  |
|  |  | N0529 | M16.9 | Xa3gR | M15 | R |  |  |  |
|  |  | Nyu23 | M17.0 | XaIna | M15 | R |  |  |  |
|  |  | Nyu24 | M17.1 | XaYQD | M15 | R |  |  |  |
|  |  | Nyu2E | M17.2 | XE1DD | M15 | R |  |  |  |
|  |  | N0545 | M17.3 | XE1DV | M15 | R |  |  |  |
|  |  | N05z5 | M17.4 | XE1Gm | M15 | A |  |  |  |
|  |  | N05zJ | M17.5 | XSDGs | M15 | R |  |  |  |
|  |  | N051B | M17.6 | 2G26. | M151 | G |  |  |  |
|  |  | Nyu25 | M17.8 | N0507 | M151 | E |  |  |  |
|  |  | N052A | M17.9 | XaBzh | M151 | G |  |  |  |
|  |  | N052C | M18.0 | XM05w | M151 | G |  |  |  |
|  |  | Nyu26 | M18.1 | N0503 | M152 | E |  |  |  |
|  |  | Nyu27 | M18.2 | X76G5 | M152 | G |  |  |  |
|  |  | Nyu28 | M18.3 | N0505 | M153 | G |  |  |  |
|  |  | N05zL | M18.4 | N0506 | M154 | G |  |  |  |
|  |  | N051C | M18.5 | XaBmY | M154 | R |  |  |  |
|  |  | Nyu29 | M18.6 | Nyu20 | M158 | E |  |  |  |
|  |  | N052B | M18.7 | N050. | M159 | D |  |  |  |
|  |  | Nyu2A | M18.8 | N0500 | M159 | G |  |  |  |
|  |  | Nyu2B | M18.9 | N0502 | M159 | G |  |  |  |
|  |  | Nyu2C |  | N050z | M159 | G |  |  |  |
|  |  | N0539 |  | N054z | M159 | G |  |  |  |
|  |  |  |  | X7038 | M159 | R |  |  |  |
|  |  |  |  | XE1DW | M159 | G |  |  |  |
|  |  |  |  | XE1Gm | M159 | D |  |  |  |
|  |  |  |  | N053. | M16 | R |  |  |  |
|  |  |  |  | X703K | M16 | A |  |  |  |
|  |  |  |  | X7041 | M16 | R |  |  |  |
|  |  |  |  | XaIna | M16 | R |  |  |  |
|  |  |  |  | XE1DD | M16 | R |  |  |  |
|  |  |  |  | XE1DV | M16 | R |  |  |  |
|  |  |  |  | XE1Gm | M16 | R |  |  |  |
|  |  |  |  | N0519 | M160 | D |  |  |  |
|  |  |  |  | N0515 | M161 | D |  |  |  |
|  |  |  |  | Nyu21 | M161 | E |  |  |  |
|  |  |  |  | N051A | M162 | D |  |  |  |
|  |  |  |  | XE1DX | M162 | R |  |  |  |
|  |  |  |  | Nyu22 | M163 | E |  |  |  |
|  |  |  |  | XE1DX | M163 | R |  |  |  |
|  |  |  |  | N0529 | M164 | D |  |  |  |
|  |  |  |  | N061. | M164 | R |  |  |  |
|  |  |  |  | XE1DX | M164 | R |  |  |  |
|  |  |  |  | XE1Go | M164 | R |  |  |  |
|  |  |  |  | N061. | M165 | R |  |  |  |
|  |  |  |  | Nyu23 | M165 | E |  |  |  |
|  |  |  |  | XE1DX | M165 | R |  |  |  |
|  |  |  |  | XE1Go | M165 | R |  |  |  |
|  |  |  |  | Nyu24 | M166 | E |  |  |  |
|  |  |  |  | XE1DX | M166 | R |  |  |  |
|  |  |  |  | Nyu2E | M167 | G |  |  |  |
|  |  |  |  | X7043 | M167 | G |  |  |  |
|  |  |  |  | XE1DX | M167 | R |  |  |  |
|  |  |  |  | XE1GV | M167 | E |  |  |  |
|  |  |  |  | N0545 | M169 | D |  |  |  |
|  |  |  |  | N05z5 | M169 | G |  |  |  |
|  |  |  |  | N05zJ | M169 | G |  |  |  |
|  |  |  |  | X7038 | M169 | R |  |  |  |
|  |  |  |  | X703K | M169 | D |  |  |  |
|  |  |  |  | XE1De | M169 | D |  |  |  |
|  |  |  |  | N053. | M17 | R |  |  |  |
|  |  |  |  | X703L | M17 | A |  |  |  |
|  |  |  |  | X7041 | M17 | R |  |  |  |
|  |  |  |  | XaIna | M17 | R |  |  |  |
|  |  |  |  | XaYQD | M17 | A |  |  |  |
|  |  |  |  | XE1DD | M17 | R |  |  |  |
|  |  |  |  | XE1Df | M17 | R |  |  |  |
|  |  |  |  | XE1DV | M17 | R |  |  |  |
|  |  |  |  | XE1DZ | M17 | R |  |  |  |
|  |  |  |  | XE1Gm | M17 | R |  |  |  |
|  |  |  |  | N051B | M170 | D |  |  |  |
|  |  |  |  | Nyu25 | M171 | G |  |  |  |
|  |  |  |  | XE1GT | M171 | E |  |  |  |
|  |  |  |  | N052A | M172 | D |  |  |  |
|  |  |  |  | N061. | M172 | R |  |  |  |
|  |  |  |  | XE1Go | M172 | R |  |  |  |
|  |  |  |  | N061. | M173 | R |  |  |  |
|  |  |  |  | Nyu26 | M173 | E |  |  |  |
|  |  |  |  | XaC1I | M173 | G |  |  |  |
|  |  |  |  | XE1Go | M173 | R |  |  |  |
|  |  |  |  | Nyu27 | M174 | E |  |  |  |
|  |  |  |  | Nyu28 | M175 | G |  |  |  |
|  |  |  |  | XE1GU | M175 | E |  |  |  |
|  |  |  |  | N05zL | M179 | G |  |  |  |
|  |  |  |  | X7038 | M179 | R |  |  |  |
|  |  |  |  | X703L | M179 | D |  |  |  |
|  |  |  |  | XaYQD | M179 | D |  |  |  |
|  |  |  |  | N053. | M18 | R |  |  |  |
|  |  |  |  | N0534 | M18 | R |  |  |  |
|  |  |  |  | N0535 | M18 | R |  |  |  |
|  |  |  |  | N0539 | M18 | A |  |  |  |
|  |  |  |  | N05zE | M18 | A |  |  |  |
|  |  |  |  | X703E | M18 | A |  |  |  |
|  |  |  |  | X703F | M18 | A |  |  |  |
|  |  |  |  | X7041 | M18 | R |  |  |  |
|  |  |  |  | Xa3gQ | M18 | R |  |  |  |
|  |  |  |  | XaIna | M18 | R |  |  |  |
|  |  |  |  | XE1DD | M18 | R |  |  |  |
|  |  |  |  | XE1DV | M18 | R |  |  |  |
|  |  |  |  | XE1DY | M18 | R |  |  |  |
|  |  |  |  | XE1Gm | M18 | R |  |  |  |
|  |  |  |  | N051C | M180 | D |  |  |  |
|  |  |  |  | Nyu29 | M181 | E |  |  |  |
|  |  |  |  | N052B | M182 | D |  |  |  |
|  |  |  |  | N061. | M182 | R |  |  |  |
|  |  |  |  | XE1Go | M182 | R |  |  |  |
|  |  |  |  | N061. | M183 | R |  |  |  |
|  |  |  |  | Nyu2A | M183 | E |  |  |  |
|  |  |  |  | XE1Go | M183 | R |  |  |  |
|  |  |  |  | Nyu2B | M184 | E |  |  |  |
|  |  |  |  | Nyu2C | M185 | E |  |  |  |
|  |  |  |  | N0539 | M189 | D |  |  |  |
|  |  |  |  | N05zE | M189 | D |  |  |  |
|  |  |  |  | X7038 | M189 | R |  |  |  |
|  |  |  |  | X703E | M189 | D |  |  |  |
|  |  |  |  | X703F | M189 | D |  |  |  |

*Supplementary Table S2. List of Read v2 and v3 codes mapped to ICD-10 codes for chronic overlapping pain conditions (COPCs)*

|  | GVIF | Df | GVIF^(1/(2*Df)) |
| --- | --- | --- | --- |
| Number of baseline CNS-driven symptoms | 1.058781397 | 3 | 1.009565227 |
| Age | 1.083928759 | 1 | 1.041118994 |
| Sex | 1.083662128 | 1 | 1.040990936 |
| Ethnicity | 1.034146633 | 1 | 1.016930004 |
| Townsend Deprivation Index | 1.043283594 | 1 | 1.021412548 |
| University Degree | 1.043106683 | 1 | 1.021325944 |
| Body Mass Index | 1.047525583 | 1 | 1.023486973 |
| Tobacco Use | 1.082908022 | 2 | 1.020112085 |
| Alcohol consumption | 1.108326239 | 3 | 1.017289595 |

Supplementary Table S3. Variance inflation factors (VIF) for baseline variables included in the model, with degrees of freedom (Df) and adjusted generalised VIF values. Values closer to 1 indicate low multicollinearity, suggesting that the baseline CNS-driven symptoms and demographic/lifestyle variables included in the model do not exhibit significant multicollinearity.

|  | **Total** | **Unavailable Primary Care Data** | **Available Primary Care Data** | **P** |
| --- | --- | --- | --- | --- |
|  | (N=496545) | (N=269072) | (N=227473) |  |
| **Age (years)** |  |  |  |  |
| Mean (SD) | 56.5 (8.09) | 56.5 (8.11) | 56.5 (8.06) | 0.366 |
| **Sex** |  |  |  |  |
| Female | 270273 (54 %) | 145858 (54 %) | 124415 (55 %) | 0.0035 |
| Male | 226272 (46 %) | 123214 (46 %) | 103058 (45 %) |  |
| **Ethicity** |  |  |  |  |
| Non-White | 25435 (5 %) | 15437 (6 %) | 9998 (4 %) | <0.001 |
| White | 469278 (95 %) | 252538 (94 %) | 216740 (95 %) |  |
| Missing | 1832 (0.4%) | 1097 (0.4%) | 735 (0.3%) |  |
| **Townsend Deprivation Index** |  |  |  |  |
| Mean (SD) | -1.32 (3.08) | -1.29 (3.13) | -1.35 (3.02) | <0.001 |
| Missing | 614 (0.1%) | 275 (0.1%) | 339 (0.1%) |  |
| **University Degree** |  |  |  |  |
| Degree | 161712 (33 %) | 88799 (33 %) | 72913 (32 %) | <0.001 |
| No Degree | 329624 (66 %) | 177446 (66 %) | 152178 (67 %) |  |
| Missing | 5209 (1.0%) | 2827 (1.1%) | 2382 (1.0%) |  |
| **Tobacco Use** |  |  |  |  |
| Never | 270539 (54 %) | 146139 (54 %) | 124400 (55 %) | 0.164 |
| Previous | 171778 (35 %) | 93402 (35 %) | 78376 (34 %) |  |
| Current | 52257 (11 %) | 28420 (11 %) | 23837 (10 %) |  |
| Missing | 1971 (0.4%) | 1111 (0.4%) | 860 (0.4%) |  |
| **Alcohol Use** |  |  |  |  |
| Never | 39263 (8 %) | 20981 (8 %) | 18282 (8 %) | <0.001 |
| Rarely | 112499 (23 %) | 60949 (23 %) | 51550 (23 %) |  |
| Weekly | 242990 (49 %) | 130509 (49 %) | 112481 (49 %) |  |
| Daily | 101229 (20 %) | 56308 (21 %) | 44921 (20 %) |  |
| Missing | 564 (0.1%) | 325 (0.1%) | 239 (0.1%) |  |
| **Body Mass Index (kg/m2)** |  |  |  |  |
| Mean (SD) | 27.4 (4.80) | 27.3 (4.78) | 27.5 (4.82) | <0.001 |
| Missing | 2346 (0.5%) | 1294 (0.5%) | 1052 (0.5%) |  |
| **Chronic Pain** |  |  |  |  |
| No chronic pain | 279314 (56 %) | 152204 (57 %) | 127110 (56 %) | <0.001 |
| Chronic pain | 216050 (44 %) | 116246 (43 %) | 99804 (44 %) |  |
| Missing | 1181 (0.2%) | 622 (0.2%) | 559 (0.2%) |  |
| **Recent Pain** |  |  |  |  |
| No recent pain | 195698 (39 %) | 106475 (40 %) | 89223 (39 %) | 0.044 |
| Recent pain | 299666 (60 %) | 161975 (60 %) | 137691 (61 %) |  |
| Missing | 1181 (0.2%) | 622 (0.2%) | 559 (0.2%) |  |
| **Executive function, Z** |  |  |  |  |
| Mean (SD) | -0.000000000000000154 (1.00) | 0.000890 (1.00) | -0.00105 (0.998) | 0.792 |
| **Insomnia** |  |  |  |  |
| Never/rarely | 119569 (24 %) | 65787 (24 %) | 53782 (24 %) | <0.001 |
| Sometimes | 236374 (48 %) | 128355 (48 %) | 108019 (47 %) |  |
| Usually | 140033 (28 %) | 74616 (28 %) | 65417 (29 %) |  |
| Missing | 569 (0.1%) | 314 (0.1%) | 255 (0.1%) |  |
| **Depression/Anxiety** |  |  |  |  |
| Mean (SD) | 0.346 (0.476) | 0.337 (0.473) | 0.356 (0.479) | <0.001 |
| Missing | 1146 (0.2%) | 655 (0.2%) | 491 (0.2%) |  |
| **No. Baseline CNS-driven symptoms** |  |  |  |  |
| 0 | 186748 (38 %) | 102983 (38 %) | 83765 (37 %) | <0.001 |
| 1 | 198469 (40 %) | 107338 (40 %) | 91131 (40 %) |  |
| 2 | 93049 (19 %) | 49162 (18 %) | 43887 (19 %) |  |
| 3 | 16736 (3 %) | 8723 (3 %) | 8013 (4 %) |  |
| Missing | 1543 (0.3%) | 866 (0.3%) | 677 (0.3%) |  |

Supplementary Table S4. Baseline characteristics for UKB participants according to availability of linked primary care record data. SD, standard deviation.

|  |  | **Number of baseline CNS-driven symptoms** | | | |
| --- | --- | --- | --- | --- | --- |
|  | **Total** | **0** | **1** | **2** | **3** |
|  | (N=70691) | (N=33701) | (N=26912) | (N=8976) | (N=1102) |
| **Age (years)** |  |  |  |  |  |
| **Mean (SD)** | 56.1 (7.65) | 55.0 (7.85) | 56.8 (7.46) | 57.9 (6.85) | 59.3 (6.30) |
| **Sex** |  |  |  |  |  |
| **Female** | 38930 (55 %) | 16159 (48 %) | 15886 (59 %) | 6094 (68 %) | 791 (72 %) |
| **Male** | 31761 (45 %) | 17542 (52 %) | 11026 (41 %) | 2882 (32 %) | 311 (28 %) |
| **Ethnicity** |  |  |  |  |  |
| **Non-White** | 1705 (2 %) | 865 (3 %) | 653 (2 %) | 170 (2 %) | 17 (2 %) |
| **White** | 68986 (98 %) | 32836 (97 %) | 26259 (98 %) | 8806 (98 %) | 1085 (98 %) |
| **Townsend Deprivation Index** |  |  |  |  |  |
| **Mean (SD)** | -1.81 (2.76) | -1.89 (2.73) | -1.78 (2.77) | -1.64 (2.82) | -1.49 (2.82) |
| **University Degree** |  |  |  |  |  |
| **Degree** | 34476 (49 %) | 17354 (51 %) | 12712 (47 %) | 3982 (44 %) | 428 (39 %) |
| **No Degree** | 36215 (51 %) | 16347 (49 %) | 14200 (53 %) | 4994 (56 %) | 674 (61 %) |
| **Tobacco Use** |  |  |  |  |  |
| **Prefer not to answer** | 0 (0 %) | 0 (0 %) | 0 (0 %) | 0 (0 %) | 0 (0 %) |
| **Never** | 42633 (60 %) | 21363 (63 %) | 15795 (59 %) | 4889 (54 %) | 586 (53 %) |
| **Previous** | 23711 (34 %) | 10430 (31 %) | 9391 (35 %) | 3450 (38 %) | 440 (40 %) |
| **Current** | 4347 (6 %) | 1908 (6 %) | 1726 (6 %) | 637 (7 %) | 76 (7 %) |
| **Alcohol Use** |  |  |  |  |  |
| **Never** | 3293 (5 %) | 1434 (4 %) | 1299 (5 %) | 492 (5 %) | 68 (6 %) |
| **Rarely** | 12648 (18 %) | 5710 (17 %) | 4963 (18 %) | 1738 (19 %) | 237 (22 %) |
| **Weekly** | 37269 (53 %) | 18361 (54 %) | 13986 (52 %) | 4410 (49 %) | 512 (46 %) |
| **Daily** | 17481 (25 %) | 8196 (24 %) | 6664 (25 %) | 2336 (26 %) | 285 (26 %) |
| **Body Mass Index (kg/m2)** |  |  |  |  |  |
| **Mean (SD)** | 26.2 (4.17) | 26.2 (4.10) | 26.2 (4.18) | 26.3 (4.36) | 26.4 (4.48) |
| **Cognition (Z-score)** |  |  |  |  |  |
| **Mean (SD)** | 0.137 (0.912) | 0.509 (0.525) | -0.0620 (1.00) | -0.487 (1.07) | -1.29 (0.729) |
| **Insomnia** |  |  |  |  |  |
| **Prefer not to answer** | 0 (0 %) | 0 (0 %) | 0 (0 %) | 0 (0 %) | 0 (0 %) |
| **Never/rarely** | 21794 (31 %) | 14200 (42 %) | 6753 (25 %) | 841 (9 %) | 0 (0 %) |
| **Sometimes** | 34153 (48 %) | 19501 (58 %) | 12648 (47 %) | 2004 (22 %) | 0 (0 %) |
| **Usually** | 14744 (21 %) | 0 (0 %) | 7511 (28 %) | 6131 (68 %) | 1102 (100 %) |
| **Depression/Anxiety** |  |  |  |  |  |
| **No mood disturbance** | 51388 (73 %) | 33701 (100 %) | 15636 (58 %) | 2051 (23 %) | 0 (0 %) |
| **Mood disturbance** | 19303 (27 %) | 0 (0 %) | 11276 (42 %) | 6925 (77 %) | 1102 (100 %) |
| **Follow-up Time (years)** |  |  |  |  |  |
| **Mean (SD)** | 10.0 (0.878) | 10.1 (0.875) | 10.0 (0.883) | 9.98 (0.875) | 9.94 (0.849) |

Supplementary Table S5. Baseline characteristics of participants according to number of symptoms (sleep disturbance, mood disturbance, cognitive dysfunction). SD, standard deviation.

|  | **Total** | **No Pain Questionnaire** | **Completed Pain Questionnaire** | **P-value** |
| --- | --- | --- | --- | --- |
|  | (N=496546) | (N=329812) | (N=166734) |  |
| **Age (years)** |  |  |  |  |
| **Mean (SD)** | 56.5 (8.09) | 56.9 (8.26) | 55.7 (7.69) | <0.001 |
| **Sex** |  |  |  |  |
| **Female** | 270273 (54 %) | 175541 (53 %) | 94732 (57 %) | <0.001 |
| **Male** | 226273 (46 %) | 154271 (47 %) | 72002 (43 %) |  |
| **Ethnicity** |  |  |  |  |
| **Non-White** | 25435 (5 %) | 20628 (6 %) | 4807 (3 %) | <0.001 |
| **White** | 469279 (95 %) | 307868 (93 %) | 161411 (97 %) |  |
| **Missing** | 1832 (0.4%) | 1316 (0.4%) | 516 (0.3%) |  |
| **Townsend Deprivation Index** |  |  |  |  |
| **Mean (SD)** | -1.32 (3.08) | -1.13 (3.18) | -1.69 (2.84) | <0.001 |
| **Missing** | 614 (0.1%) | 427 (0.1%) | 187 (0.1%) |  |
| **University Degree** |  |  |  |  |
| **Degree** | 161713 (33 %) | 85902 (26 %) | 75811 (45 %) | <0.001 |
| **No Degree** | 329624 (66 %) | 239234 (73 %) | 90390 (54 %) |  |
| **Missing** | 5209 (1.0%) | 4676 (1.4%) | 533 (0.3%) |  |
| **Tobacco Use** |  |  |  |  |
| **Never** | 270539 (54 %) | 174167 (53 %) | 96372 (58 %) | <0.001 |
| **Previous** | 171779 (35 %) | 113603 (34 %) | 58176 (35 %) |  |
| **Current** | 52257 (11 %) | 40407 (12 %) | 11850 (7 %) |  |
| **Missing** | 1971 (0.4%) | 1635 (0.5%) | 336 (0.2%) |  |
| **Alcohol Use** |  |  |  |  |
| **Never** | 39263 (8 %) | 30138 (9 %) | 9125 (5 %) | <0.001 |
| **Rarely** | 112499 (23 %) | 78784 (24 %) | 33715 (20 %) |  |
| **Weekly** | 242990 (49 %) | 157490 (48 %) | 85500 (51 %) |  |
| **Daily** | 101230 (20 %) | 62892 (19 %) | 38338 (23 %) |  |
| **Missing** | 564 (0.1%) | 508 (0.2%) | 56 (0.0%) |  |
| **Body Mass Index (kg/m2)** |  |  |  |  |
| **Mean (SD)** | 27.4 (4.80) | 27.8 (4.88) | 26.8 (4.57) | <0.001 |
| **Missing** | 2346 (0.5%) | 1986 (0.6%) | 360 (0.2%) |  |
| **Cognition (Z-score)** |  |  |  |  |
| **Mean (SD)** | -0.0000000000000000112 (1.00) | -0.0703 (1.03) | 0.139 (0.913) | <0.001 |
| **Insomnia** |  |  |  |  |
| **Never/rarely** | 119570 (24 %) | 76657 (23 %) | 42913 (26 %) | <0.001 |
| **Sometimes** | 236374 (48 %) | 156563 (47 %) | 79811 (48 %) |  |
| **Usually** | 140033 (28 %) | 96108 (29 %) | 43925 (26 %) |  |
| **Missing** | 569 (0.1%) | 484 (0.1%) | 85 (0.1%) |  |
| **Depression/Anxiety** |  |  |  |  |
| **No mood disturbance** | 323947 (65 %) | 213363 (65 %) | 110584 (66 %) | <0.001 |
| **Mood disturbance** | 171453 (35 %) | 115445 (35 %) | 56008 (34 %) |  |
| **Missing** | 1146 (0.2%) | 1004 (0.3%) | 142 (0.1%) |  |
| **Number of baseline symptoms** |  |  |  |  |
| **0** | 186749 (38 %) | 117630 (36 %) | 69119 (41 %) | <0.001 |
| **1** | 198469 (40 %) | 132648 (40 %) | 65821 (39 %) |  |
| **2** | 93049 (19 %) | 65538 (20 %) | 27511 (16 %) |  |
| **3** | 16736 (3 %) | 12672 (4 %) | 4064 (2 %) |  |
| **Missing** | 1543 (0.3%) | 1324 (0.4%) | 219 (0.1%) |  |

*Supplementary Table S6. Baseline characteristics for UK Biobank participants who completed the follow-up pain questionnaire compared to those who did not.*

| **Diagnosis** | **Count** | **%** |
| --- | --- | --- |
| No COPC Diagnosis | 57,894 |  |
| Low back pain | 5,009 | 61.95 |
| Chronic Fatigue Syndrome | 1,514 | 18.72 |
| Irritable Bowel Syndrome | 616 | 7.62 |
| Migraine | 530 | 6.55 |
| Non-migraine headache | 166 | 2.05 |
| Temporo-Mandibular Joint syndrome | 99 | 1.22 |
| Vulvodynia | 71 | 0.88 |
| Prostatitis | 46 | 0.57 |
| Endometriosis | 25 | 0.31 |
| Cystitis | 10 | 0.12 |

Supplementary Table S7. First chronic primary pain condition. Total number of first diagnoses was 8,086.

|  | **Unadjusted** |  |  | **Fully adjusted** |  |  |
| --- | --- | --- | --- | --- | --- | --- |
|  | **HR** | **95%CI** | **P** | **HR** | **95%CI** | **P** |
| **No. baseline CNS-driven symptoms** | - | - | - | - | - | - |
| 0 | - | - | - | - | - | - |
| 1 | 1.2 | 1.14, 1.26 | <0.001 | 1.16 | 1.10, 1.22 | <0.001 |
| 2+ | 1.43 | 1.34, 1.52 | <0.001 | 1.35 | 1.27, 1.44 | <0.001 |
| Female sex | - | - | - | 1.18 | 1.13, 1.23 | <0.001 |
| Age group (years) | - | - | - | - | - | - |
| <55 | - | - | - | - | - | - |
| 55-60 | - | - | - | 0.884 | 0.831, 0.940 | <0.001 |
| 60-65 | - | - | - | 0.944 | 0.892, 1.00 | 0.049 |
| >65 | - | - | - | 0.99 | 0.926, 1.06 | 0.8 |
| Non-white ethnicity | - | - | - | 1.37 | 1.23, 1.53 | <0.001 |
| **TDI (Quintile)** | - | - | - | - | - | - |
| Q1 | - | - | - | - | - | - |
| Q2 | - | - | - | 0.977 | 0.910, 1.05 | 0.5 |
| Q3 | - | - | - | 1.01 | 0.939, 1.08 | 0.8 |
| Q4 | - | - | - | 1.02 | 0.950, 1.09 | 0.6 |
| Q5 | - | - | - | 1.07 | 0.996, 1.15 | 0.066 |
| Degree | - | - | - | 0.788 | 0.751, 0.827 | <0.001 |
| Body Mass Index (kg/m2) | - | - | - | 1.01 | 1.01, 1.02 | <0.001 |
| **Tobacco Use** | - | - | - | - | - | - |
| Never | - | - | - | - | - | - |
| Previous | - | - | - | 1.11 | 1.06, 1.17 | <0.001 |
| Current | - | - | - | 1.15 | 1.07, 1.25 | <0.001 |
| **Alcohol Use** | - | - | - | - | - | - |
| Never | - | - | - | - | - | - |
| Rarely | - | - | - | 0.972 | 0.882, 1.07 | 0.6 |
| Weekly | - | - | - | 0.932 | 0.851, 1.02 | 0.13 |
| Daily | - | - | - | 0.928 | 0.841, 1.03 | 0.14 |

Supplementary Table S8. Full results of Cox regression for association between baseline symptoms and time to first diagnosis of a chronic primary pain condition.

|  | **Pain category at follow-up;**  **OR (95%CI)** | | | |
| --- | --- | --- | --- | --- |
| **Variable** | **CP- NP+** | **CP+ NP-** | **CP+ NP+** | **CP+ NP++** |
| **Unadjusted model** |  |  |  |  |
| One symptom | 1.46 (1.39, 1.53) <0.001 | 1.02 (0.96, 1.08) 0.471 | 1.46 (1.40, 1.52) <0.001 | 1.75 (1.63, 1.87) <0.001 |
| Two symptoms | 2.33 (2.18, 2.50) <0.001 | 1.07 (0.97, 1.18) 0.164 | 2.27 (2.14, 2.41) <0.001 | 3.03 (2.77, 3.32) <0.001 |
| Three symptoms | 2.96 (2.47, 3.54) <0.001 | 1.13 (0.86, 1.49) 0.390 | 2.97 (2.54, 3.48) <0.001 | 4.55 (3.67, 5.65) <0.001 |
| **Fully adjusted model** |  |  |  |  |
| One symptom | 1.48 (1.41, 1.55) <0.001 | 1.01 (0.95, 1.07) 0.708 | 1.38 (1.32, 1.44) <0.001 | 1.65 (1.54, 1.78) <0.001 |
| Two symptoms | 2.37 (2.21, 2.54) <0.001 | 1.05 (0.95, 1.16) 0.313 | 2.06 (1.94, 2.18) <0.001 | 2.73 (2.48, 3.00) <0.001 |
| Three symptoms | 3.08 (2.57, 3.69) <0.001 | 1.11 (0.84, 1.47) 0.459 | 2.62 (2.24, 3.07) <0.001 | 3.99 (3.20, 4.96) <0.001 |
| Age (years) | 0.97 (0.97, 0.98) <0.001 | 1.00 (1.00, 1.01) 0.146 | 1.00 (0.99, 1.00) 0.017 | 0.99 (0.98, 0.99) <0.001 |
| Female | 1.38 (1.32, 1.45) <0.001 | 1.11 (1.05, 1.17) <0.001 | 1.60 (1.53, 1.66) <0.001 | 1.58 (1.48, 1.69) <0.001 |
| White | 1.42 (1.22, 1.66) <0.001 | 1.08 (0.90, 1.30) 0.432 | 1.20 (1.06, 1.36) 0.003 | 0.88 (0.73, 1.06) 0.171 |
| Townsend Deprivation Index | 1.01 (1.00, 1.02) 0.003 | 0.98 (0.97, 0.99) 0.001 | 1.00 (1.00, 1.01) 0.484 | 1.02 (1.01, 1.04) <0.001 |
| University Degree | 1.07 (1.02, 1.12) 0.004 | 1.15 (1.09, 1.22) <0.001 | 0.91 (0.87, 0.94) <0.001 | 0.77 (0.72, 0.82) <0.001 |
| BMI (kg/m2) | 1.02 (1.01, 1.03) <0.001 | 1.02 (1.01, 1.03) <0.001 | 1.05 (1.05, 1.06) <0.001 | 1.08 (1.07, 1.09) <0.001 |
| Ex-smoker | 1.17 (1.12, 1.23) <0.001 | 1.11 (1.04, 1.18) 0.001 | 1.26 (1.21, 1.31) <0.001 | 1.46 (1.36, 1.56) <0.001 |
| Current smoker | 1.14 (1.04, 1.25) 0.006 | 1.01 (0.90, 1.15) 0.834 | 1.15 (1.06, 1.24) 0.001 | 1.71 (1.52, 1.93) <0.001 |
| Alcohol (Rarely) | 1.08 (0.96, 1.21) 0.203 | 1.11 (0.95, 1.30) 0.176 | 1.07 (0.97, 1.18) 0.158 | 1.02 (0.87, 1.19) 0.831 |
| Alcohol (Weekly) | 0.97 (0.88, 1.09) 0.642 | 1.18 (1.02, 1.36) 0.022 | 0.99 (0.91, 1.09) 0.898 | 0.88 (0.76, 1.02) 0.091 |
| Alcohol (Daily) | 0.99 (0.89, 1.11) 0.921 | 1.26 (1.08, 1.46) 0.003 | 1.05 (0.95, 1.15) 0.349 | 0.89 (0.76, 1.04) 0.136 |

Supplementary Table S9. Results for multinomial regression analysis of association between baseline symptoms and pain status at follow-up. Reference category is CP- NP- (no chronic pain and no nociplastic symptoms at follow-up). OR, odds ratio. CI, confidence interval. CP, chronic pain. NP, nociplastic pain.

|  | **Pain category at follow-up;**  **OR (95%CI)** | | | |
| --- | --- | --- | --- | --- |
| Variable | CP- NP+ | CP+ NP- | CP+ NP+ | CP+ NP++ |
| **Unadjusted model** |  |  |  |  |
| One symptom | 1.32 (1.22, 1.42) <0.001 | 1.04 (0.99, 1.09) 0.150 | 1.37 (1.32, 1.43) <0.001 | 1.55 (1.45, 1.67) <0.001 |
| Two symptoms | 1.80 (1.63, 2.00) <0.001 | 1.15 (1.06, 1.24) <0.001 | 1.88 (1.78, 1.99) <0.001 | 2.24 (2.04, 2.46) <0.001 |
| Three symptoms | 2.15 (1.67, 2.77) <0.001 | 1.25 (1.02, 1.53) 0.033 | 2.29 (1.99, 2.64) <0.001 | 3.00 (2.41, 3.72) <0.001 |
| **Fully adjusted model** |  |  |  |  |
| One symptom | 1.33 (1.23, 1.44) <0.001 | 1.02 (0.97, 1.08) 0.413 | 1.29 (1.23, 1.34) <0.001 | 1.47 (1.37, 1.58) <0.001 |
| Two symptoms | 1.82 (1.64, 2.02) <0.001 | 1.12 (1.03, 1.21) 0.006 | 1.68 (1.58, 1.78) <0.001 | 2.01 (1.83, 2.21) <0.001 |
| Three symptoms | 2.24 (1.74, 2.89) <0.001 | 1.21 (0.98, 1.49) 0.070 | 1.97 (1.70, 2.27) <0.001 | 2.61 (2.09, 3.25) <0.001 |
| Age (years) | 0.97 (0.96, 0.97) <0.001 | 1.00 (1.00, 1.01) 0.050 | 1.00 (1.00, 1.00) 0.174 | 1.00 (0.99, 1.00) 0.077 |
| Female | 1.55 (1.44, 1.66) <0.001 | 1.13 (1.08, 1.19) <0.001 | 1.56 (1.50, 1.62) <0.001 | 1.41 (1.32, 1.51) <0.001 |
| White | 1.50 (1.17, 1.91) 0.001 | 1.13 (0.96, 1.33) 0.145 | 1.08 (0.96, 1.22) 0.210 | 0.86 (0.71, 1.05) 0.132 |
| Townsend Deprivation Index | 1.01 (1.00, 1.02) 0.076 | 0.99 (0.98, 1.00) 0.003 | 1.00 (0.99, 1.01) 0.867 | 1.02 (1.01, 1.03) 0.003 |
| University Degree | 1.08 (1.01, 1.16) 0.035 | 1.12 (1.07, 1.18) <0.001 | 0.87 (0.84, 0.90) <0.001 | 0.78 (0.73, 0.83) <0.001 |
| BMI (kg/m2) | 1.03 (1.02, 1.04) <0.001 | 1.02 (1.02, 1.03) <0.001 | 1.05 (1.05, 1.05) <0.001 | 1.08 (1.07, 1.08) <0.001 |
| Ex-smoker | 1.24 (1.15, 1.33) <0.001 | 1.12 (1.06, 1.18) <0.001 | 1.23 (1.19, 1.28) <0.001 | 1.42 (1.33, 1.53) <0.001 |
| Current smoker | 1.06 (0.91, 1.23) 0.457 | 0.97 (0.87, 1.08) 0.583 | 1.15 (1.06, 1.24) 0.001 | 1.62 (1.43, 1.83) <0.001 |
| Alcohol (Rarely) | 0.92 (0.77, 1.09) 0.308 | 1.06 (0.93, 1.21) 0.372 | 1.04 (0.94, 1.14) 0.466 | 0.99 (0.84, 1.16) 0.885 |
| Alcohol (Weekly) | 0.84 (0.71, 0.98) 0.030 | 1.11 (0.99, 1.25) 0.086 | 0.97 (0.89, 1.06) 0.517 | 0.91 (0.79, 1.06) 0.246 |
| Alcohol (Daily) | 0.89 (0.75, 1.05) 0.172 | 1.20 (1.06, 1.36) 0.005 | 1.01 (0.92, 1.11) 0.769 | 0.92 (0.78, 1.07) 0.284 |

*Supplementary Table S10. Sensitivity analysis for multinomial regression analysis with items related to mood, sleep, and cognitive disturbance removed from outcome measures. BMI, body mass index. OR, odds ratio. CI, confidence interval. CP, chronic pain. NP, nociplastic pain.*

| **Pain category level** | **Exposure (Number symptoms)** | **E Value Estimate** | **E Value Lower CI** |
| --- | --- | --- | --- |
| **CP- NP+** | rf01 | 1.47877934134834 | 2.32021204754075 |
| **CP- NP+** | rf02 | 2.36808312054036 | 4.1680093815988 |
| **CP- NP+** | rf03 | 3.0799440791122 | 5.61097380065758 |
| **CP+ NP-** | rf01 | 1.01149890584357 | 1.11934662570433 |
| **CP+ NP-** | rf02 | 1.05190718172816 | 1.28557690001258 |
| **CP+ NP-** | rf03 | 1.11093649716294 | 1.46199689590611 |
| **CP+ NP+** | rf01 | 1.378533071691 | 2.10090441529125 |
| **CP+ NP+** | rf02 | 2.05747202198873 | 3.53250391970405 |
| **CP+ NP+** | rf03 | 2.61989395679687 | 4.679979000688 |
| **CP+ NP++** | rf01 | 1.65490854870816 | 2.69597231038619 |
| **CP+ NP++** | rf02 | 2.72908967801791 | 4.90137897565899 |
| **CP+ NP++** | rf03 | 3.98634337194613 | 7.4366462727235 |

Supplementary Table S11. E Value estimates for the fully adjusted multinomial regression model. CI, confidence interval. CP, chronic pain. NP, nociplastic pain.

|  | **Unadjusted** | | | **Fully adjusted** | | |
| --- | --- | --- | --- | --- | --- | --- |
| **Characteristic** | **Beta** | **95% CI** | **P** | **Beta** | **95% CI** | **P** |
| Risk Factors (Count) | - | - | - | - | - | - |
| 0 | - | - | - | - | - | - |
| 1 | 0.191 | 0.167, 0.215 | <0.001 | 0.144 | 0.119, 0.168 | <0.001 |
| 2 | 0.434 | 0.399, 0.469 | <0.001 | 0.348 | 0.313, 0.384 | <0.001 |
| 3 | 0.574 | 0.483, 0.665 | <0.001 | 0.46 | 0.370, 0.551 | <0.001 |
| Age (years) | - | - | - | 0.002 | 0.001, 0.004 | 0.006 |
| Female | - | - | - | 0.288 | 0.264, 0.311 | <0.001 |
| White | - | - | - | -0.04 | -0.114, 0.033 | 0.3 |
| Townsend Deprivation Index | - | - | - | 0.007 | 0.003, 0.011 | <0.001 |
| University Degree | - | - | - | -0.083 | -0.106, -0.061 | <0.001 |
| Body Mass Index (kg/m2) | - | - | - | 0.028 | 0.026, 0.031 | <0.001 |
| Tobacco Use | - | - | - | - | - | - |
| Never | - | - | - | - | - | - |
| Previous | - | - | - | 0.116 | 0.092, 0.141 | <0.001 |
| Current | - | - | - | 0.137 | 0.089, 0.184 | <0.001 |
| Alcohol Use (Frequency) | - | - | - | - | - | - |
| Never | - | - | - | - | - | - |
| Rarely | - | - | - | -0.029 | -0.086, 0.029 | 0.3 |
| Weekly | - | - | - | -0.072 | -0.126, -0.018 | 0.009 |
| Daily | - | - | - | -0.06 | -0.117, -0.003 | 0.037 |

Supplementary Table S12. Association between baseline symptoms and fibromyalgianess scores at follow-up. CI, confidence interval.

| **Estimate.rf01** | **E Value Estimate** | **E Value Lower CI** |
| --- | --- | --- |
| **One symptom** | 1.15435889707016 | 1.57647921898666 |
| **Two symptoms** | 1.41631093288243 | 2.18418183721457 |
| **Three symptoms** | 1.58477428046478 | 2.54744516896089 |

Supplementary Table S13. E Values for analysis 3: baseline symptoms and nociplastic pain severity at follow-up.

**
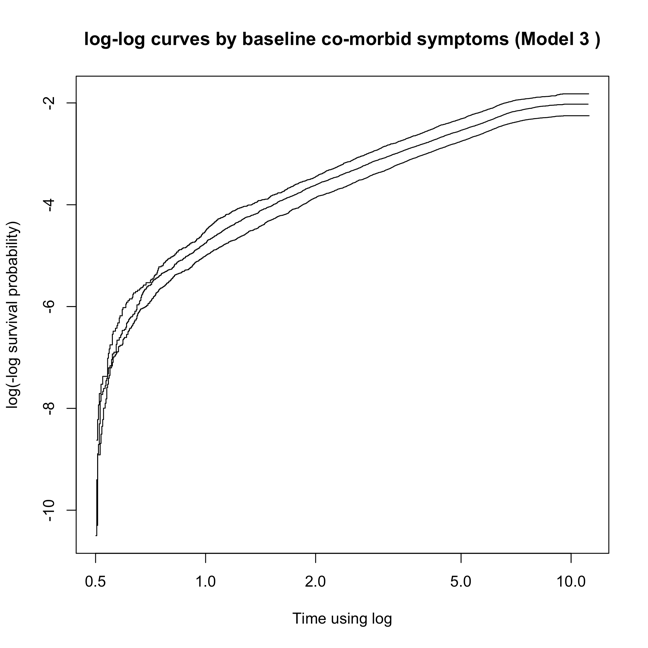

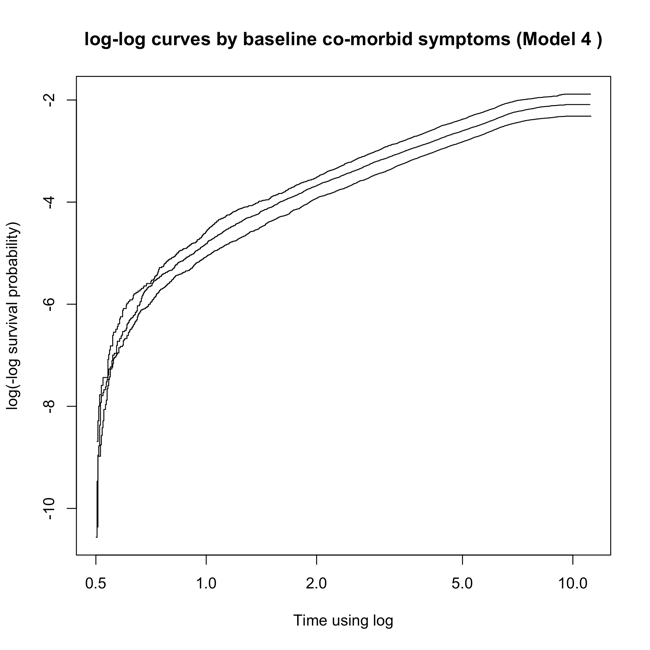

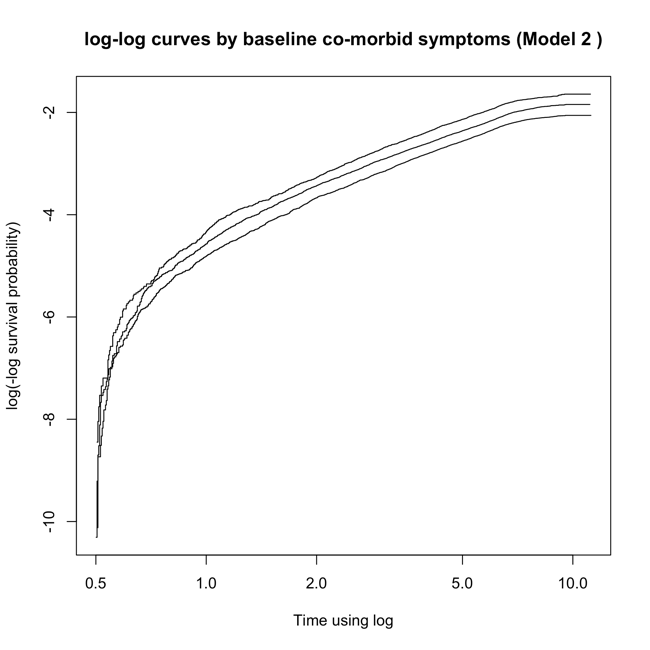

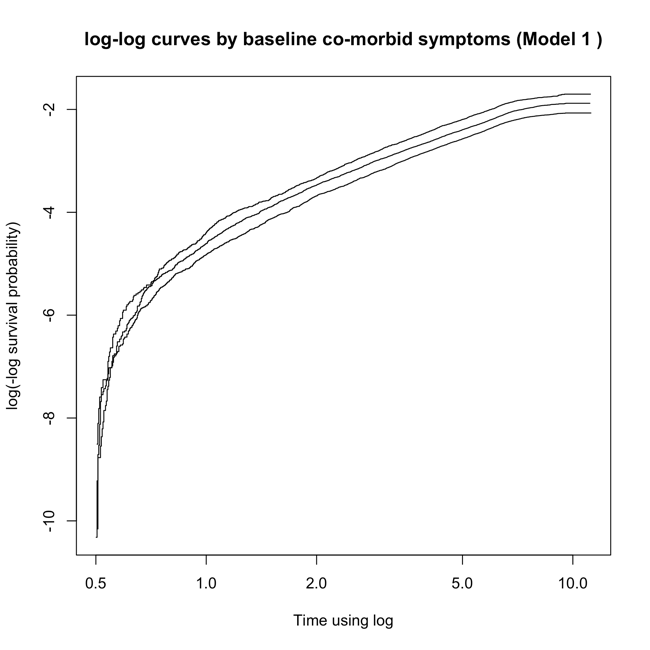
**

*Supplementary Figure S1. Log-log plots for Cox regression models.*

*We assessed the proportional hazards assumption for our survival model using visual inspection of the log-log plot and testing the Schoenfeld residuals using the `cox.zph` function in R. For the four models, visual inspection of the log-log plot revealed no major violations. The test for Schoenfeld residuals yielded a slightly significant result for the number of baseline symptoms model 1 (chi-square = 6.06, df = 2, P = 0.048). This was similar in the other three models. Considering the large sample size of our study, the Schoenfeld residuals test can be highly sensitive, and the slight significance observed may not be practically meaningful.*


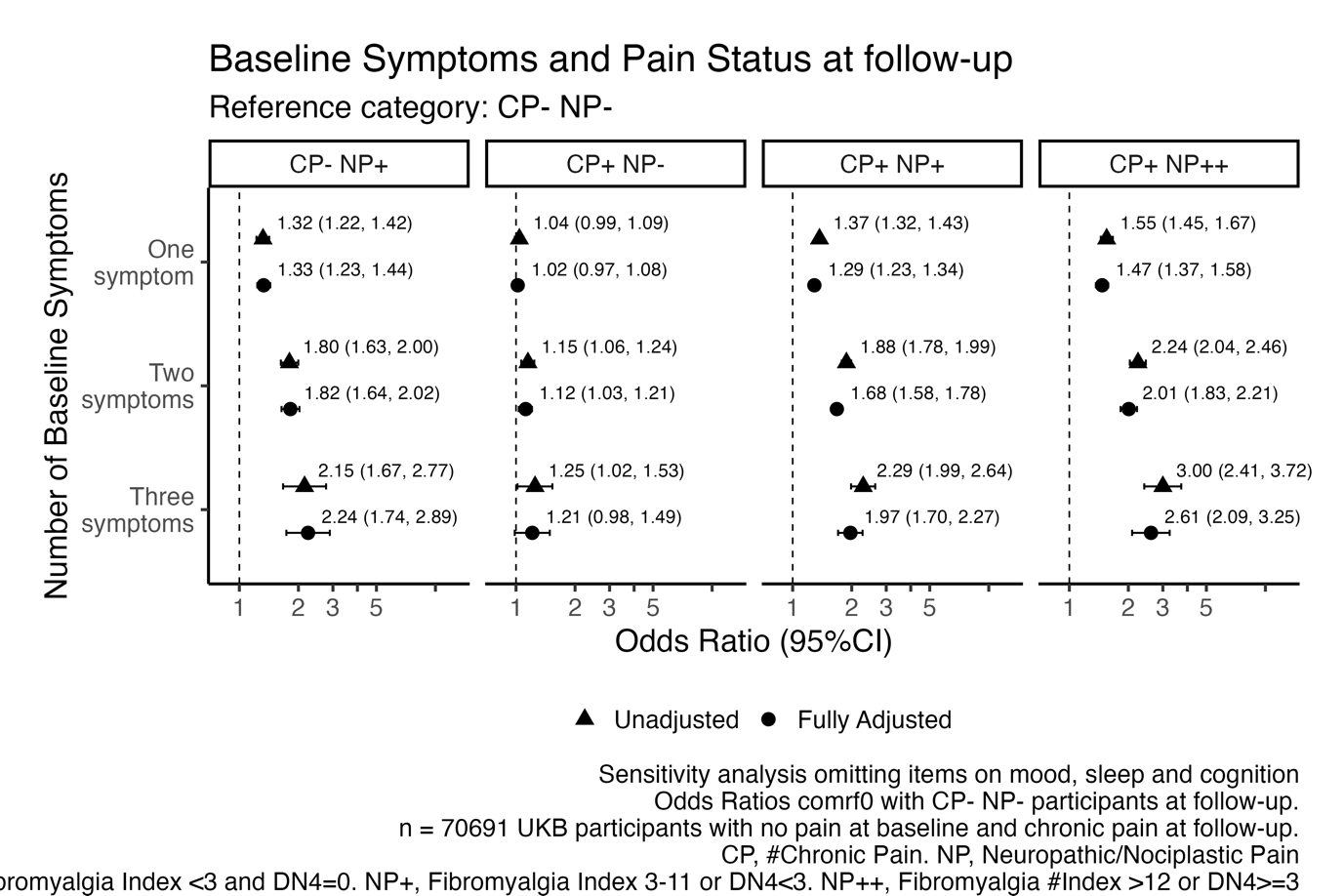


Supplementary Figure S2. Multinomial regression results. Sensitivity analysis with items on mood, sleep, and cognitive disturbance removed.
